# Effects of different treatments for type 2 diabetes mellitus on mortality of coronavirus disease from 2019 to 2021 in China: a multi-institutional retrospective study

**DOI:** 10.1186/s43556-024-00183-1

**Published:** 2024-05-17

**Authors:** Ke Xu, Wu He, Bo Yu, Kaineng Zhong, Da Zhou, Dao Wen Wang

**Affiliations:** 1 Division of Cardiology, Department of Internal Medicine, Hubei Key Laboratory of Genetics and Molecular Mechanisms of Cardiological Disorders, Tongji Medical College, Tongji Hospital, Huazhong University of Science and Technology, 1095# Jiefang Ave., Wuhan, 430030 China; 2 Hubei Provincial Health Commission, Wuhan, 430079 China

**Keywords:** COVID-19, Type 2 diabetes mellitus (T2DM), Metformin, AGIs, Insulin

## Abstract

The coronavirus disease (COVID-19) pandemic has continued for 5 years. Sporadic cases continue to occur in different locations. Type 2 diabetes mellitus (T2DM) is associated with a high risk of a poor prognosis in patients with COVID-19. Successful control of blood glucose levels can effectively decrease the risks of severe infections and mortality. However, the effects of different treatments were reported differently and even adversely. This retrospective study included 4,922 patients who have been diagnosed as COVID-19 and T2DM from 138 Hubei hospitals. The clinical characteristics and outcomes were compared and calculated their risk for death using multivariate Cox regression and Kaplan–Meier curves. After adjustment of age, sex, comorbidities, and in-hospital medications, metformin and alpha-glucosidase inhibitor (AGI) use performed lower all-cause mortality (adjusted hazard ratio [HR], 0.41; 95% confidence interval [CI]: 0.24–0.71; *p* = 0.001 for metformin; 0.53, 0.35–0.80, *p* = 0.002 for AGIs), while insulin use was associated with increased all-cause mortality (adjusted HR, 2.07, 95% CI, 1.61–2.67, *p* < 0.001). After propensity score-matched (PSM) analysis, adjusted HRs for insulin, metformin, and AGIs associated with all-cause mortality were 1.32 (95% CI, 1.03–1.81; *p* = 0.012), 0.48 (95% CI, 0.23–0.83, *p* = 0.014), and 0.59 (95% CI, 0.35–0.98, *p* = 0.05). Therefore, metformin and AGIs might be more suitable for patients with COVID-19 and T2DM while insulin might be used with caution.

**Supplementary Information:**

The online version contains supplementary material available at 10.1186/s43556-024-00183-1.

## Introduction

The coronavirus disease (COVID-19) pandemic has continued for 5 years since the first case was reported in December 2019. In the early stage, the novel coronavirus was well-known for its highly contagious and severe respiratory symptoms which bring a terrible blow to public health around the world. As time went on, the proper management has been conducted and vaccines for the virus have been developed which seems to mean that the pandemic was going to the end. It was unexpected that the virus was mutating at a very rapid rate. As of Aug 9, 2023, there have been over 700 million cases and over than six million deaths globally reported by the World Health Organization. Sporadic cases still exist in different locations, although most countries have implemented COVID-19 control.

Type 2 diabetes mellitus (T2DM) is associated with a high risk of a poor prognosis in patients with COVID-19, and patients with COVID-19 and diabetes might develop acute respiratory distress syndrome (ARDS) and severe viral infection [1–4]. Successful control of blood glucose can effectively decrease the risk of severe infection and mortality [2, 5]. Therefore, glucose control in patients with COVID-19 and T2DM is crucial to reduce the risk of complications and poor prognosis. Post-COVID-19 T2DM in the context of long COVID-19 has been paid more attention to, and how to manage these patients has become an important topic [6, 7].

In the early stages, some studies suggested that insulin treatment was prioritized over oral hypoglycemic drugs in treating patients with COVID-19 and T2DM [8, 9]. However, at that time, evidence was lacking. An association between metformin treatment and a lowered risk was proposed in many studies [10–13]. Our laboratory study found that insulin treatment in patients with T2DM significantly increased mortality and promoted infection and inflammation in different organs, contrary to common reports [14].

To determine the effect of different antidiabetic drugs on the outcomes of patients with COVID-19 and T2DM, we used the data of patients with COVID-19 from December 2019 to August 2021 in Hubei Province, China, to compare common antidiabetic drugs, such as insulin, metformin, alpha-glycosidase inhibitors, sulfonylureas, glinides, and DPP4 inhibitors. The results might provide more suggestions and evidence regarding which drugs might be a priority to manage patients with COVID-19 and T2DM or post-COVID-19 T2DM.

## Results

### Participants

**Fig. 1:**
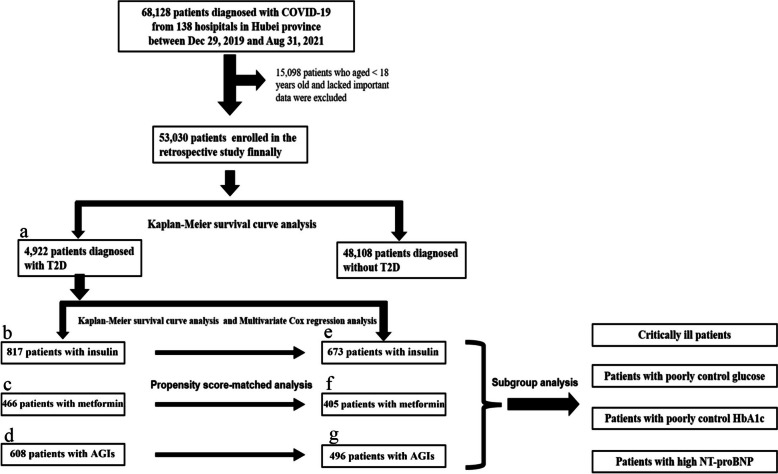
The flowchart about the process of participant enrolment and data analysis. **a** 4922 participants with a history of T2DM were classified into the disease group; **b**, **c**, **d** 817, 466 and 608 patients with T2DM who were taking insulin, metformin and AGIs during hospitalization were enrolled in the insulin group; **e**, **f**, **d** 673, 405 and 496 patients with T2DM who were taking insulin, metformin and AGIs during hospitalization were enrolled in the insulin group after PSM

Before August 31, 2021, 68,128 subjects confirmed with COVID-19 participated in our study from 138 hospitals in Hubei Province. Of these, missing data helped us exclude 15,098 patients (22.2%). In the end, the number of 53,030 patients were included in the study. Among them, 4,922 (9.28%) were diagnosed with T2DM. The median age of the 4,922 patients was 66 (58–76) years, and 53.4% of the patients were men (Table 1). Table 1 shows the other characteristics of the T2DM and non-T2DM groups and the flow chart is shown in Fig. 1. Compared to the non-T2DM group, the T2DM group had higher proportions of cardiovascular diseases, chronic obstructive pulmonary disease and chronic kidney disease.

**Table 1:** Characteristics of patients with COVID-19 in the type 2 diabetes and non-T2D groups

	Patients without diabetes (*n* = 48,108)	Patients with diabetes (*n* = 4922)	*P*-value
Gender: Male (%)	23,916 (49.7)	2630 (53.4)	< 0.001
Age, years	58.00 [45.00, 69.00]	66.00 [58.00, 76.00]	< 0.001
Age ≥ 65 years (%)	30,682 (63.8)	2116 (43.0)	< 0.001
Age<65 years (%)	17,426 (36.2)	2806 (57.0)	< 0.001
Hospitalization time (days)	13.00 [7.00, 20.00]	11.00 [7.00, 18.00]	< 0.001
COVID-19 Severity (n %)			
Mild	21,046 (43.7)	1822 (37.0)	< 0.001
Normal	15,781 (32.8)	1512 (30.7)	0.056
Severe	8967 (18.6)	1246 (25.3)	< 0.001
Critical	2314 (4.8)	342 (6.9)	< 0.001
Gender: Male (%)	23,916 (49.7)	2630 (53.4)	< 0.001
**Vital signs**			
Systolic blood pressure (mmHg)	121.00 [104.00, 132.00]	127.00 [115.00, 140.00]	< 0.001
Diastolic blood pressure (mmHg)	80.00 [72.00, 94.00]	79.00 [70.00, 90.00]	< 0.001
Respiratory rate (times per minute)	20.00 [19.00, 20.00]	20.00 [19.00, 20.00]	0.69
Pulse rate (times per minute)	80.00 [75.00, 89.00]	80.00 [74.00, 89.00]	< 0.001
Temperature (°C)	36.50 [36.30, 36.80]	36.50 [36.30, 36.70]	< 0.001
**Origin Comorbidities**			
Hypertension (%)	6660 (13.8)	3076 (62.5)	< 0.001
Hypercholesterolemia (%)	21,527 (44.7)	2340 (47.5)	0.056
Coronary heart disease (%)	1972 (4.1)	1241 (25.2)	< 0.001
COPD (%)	897 (1.9)	267 (5.4)	< 0.001
Heart failure (%)	954 (2.0)	464 (9.4)	< 0.001
Arrhythmia (%)	969 (2.0)	384 (7.8)	< 0.001
CKD (%)	653 (1.4)	392 (8.0)	< 0.001
**Laboratory results**			
** Routine blood test**			
Red blood cell count, ×10^12^/L	4.18 [3.79, 4.57]	4.13 [3.69, 4.55]	< 0.001
White cell count, ×10^9^/L	5.80 [4.60, 7.50]	6.14 [4.83, 7.89]	< 0.001
Hemoglobin g/L	126.00 [114.00, 138.00]	125.00 [112.00, 137.00]	< 0.001
Neutrophil count, ×10^9^/L	3.61 [2.68, 5.11]	3.97 [2.92, 5.60]	< 0.001
Monocyte count, ×10^9^/L	0.41 [0.30, 0.54]	0.41 [0.31, 0.55]	0.107
Platelet count, ×10^9^/L	201.00 [161.00, 241.00]	194.00 [155.00, 236.00]	< 0.001
** Blood biochemistry**			
Alanine aminotransferase, U/L	21.60 [14.00, 36.00]	20.40 [14.00, 32.50]	< 0.001
Aspartate aminotransferase, U/L	22.00 [17.00, 31.70]	21.70 [16.60, 30.70]	< 0.001
Lactate dehydrogenase, U/L	190.00 [156.00, 248.00]	187.00 [153.00, 246.00]	0.001
Total bilirubin, umol/L	10.10 [7.40, 13.80]	10.60 [7.97, 14.60]	< 0.001
Total protein, g/L	67.00 [62.30, 71.80]	67.10 [61.90, 72.10]	0.745
Globulin, g/L	28.00 [24.40, 31.90]	27.90 [24.60, 31.70]	0.939
Albumin, g/L	38.70 [34.70, 42.60]	38.90 [34.52, 42.80]	0.421
Alkaline phosphatase, U/L	67.00 [53.60, 84.60]	71.80 [57.20, 90.47]	< 0.001
Total cholesterol, mmol/L	4.11 [3.47, 4.85]	4.14 [3.45, 4.94]	0.146
Triglyceride, mmol/L	1.30 [0.95, 1.85]	1.35 [1.00, 1.90]	< 0.001
LDL, mmol/L	4.11 [3.47, 4.85]	4.14 [3.45, 4.94]	0.146
HDL, mmol/L	1.07 [0.88, 1.31]	1.06 [0.86, 1.29]	0.001
Creatinine, µmol/L	66.00 [55.00, 80.00]	69.50 [57.00, 87.00]	< 0.001
Blood urea nitrogen, mmol/L	4.56 [3.60, 5.87]	4.80 [3.80, 6.60]	< 0.001
eGFR, mL/min	100.00 [86.20, 114.35]	97.40 [81.80, 112.20]	< 0.001
Sodium, mmol/L	139.90 [137.40, 141.80]	139.20 [136.70, 141.60]	< 0.001
Potassium, mmol/L	4.02 [3.70, 4.36]	3.99 [3.67, 4.32]	< 0.001
Calcium, mmol/L	2.23 [2.11, 2.36]	2.25 [2.12, 2.37]	0.001
Lactic acid, mmol/L	1.70 [1.21, 2.30]	1.74 [1.30, 2.40]	< 0.001
** Coagulation function**			
APTT	31.40 [27.60, 35.50]	30.70 [27.10, 34.80]	< 0.001
Prothrombin time, s	12.40 [11.10, 13.60]	11.80 [10.70, 13.30]	< 0.001
Prothrombin activity, %	96.00 [85.70, 106.60]	95.00 [83.00, 106.00]	< 0.001
Thrombin time, s	16.50 [15.20, 17.80]	16.80 [15.50, 18.10]	< 0.001
International normalized ratio	1.01 [0.92, 1.11]	0.97 [0.89, 1.09]	< 0.001
Fibrinogen, mg/L	3.32 [2.58, 4.30]	3.30 [2.56, 4.22]	0.05
D-dimer, mg/L	0.46 [0.24, 0.88]	0.52 [0.29, 0.95]	< 0.001
** Cardiac function related indicators**			
NT-proBNP	98.30 [33.40, 335.00]	130.00 [40.23, 466.75]	< 0.001
hscTnI	0.04 [0.01, 3.80]	0.03 [0.01, 3.30]	0.001
CK-MB	7.00 [0.93, 12.40]	7.00 [0.90, 12.21]	0.148
** Diabetes related index**			
Glucose, mmol/L	5.64 [4.99, 7.00]	6.17 [5.17, 8.56]	< 0.001
HbA1c, %	6.20 [5.70, 7.20]	6.50 [5.80, 7.80]	< 0.001
**Outcomes**			
Acute cardiac injury	1739 (3.6)	405 (8.2)	< 0.001
Mechanical ventilation	1609 (3.3)	209 (4.2)	0.001
ECMO	174 (0.4)	22 (0.4)	0.415
Renal replacement therapy	311 (0.6)	71 (1.4)	< 0.001

Data were presented as median and interquartile range (Q1-Q3)

*Abbreviations:COPD* Chronic obstructive pulmonary disease, *CKD* Chronic kidney disease, *LDL* Low-density lipoprotein, *HDL* High-density lipoprotein, *eGFR* Estimated glomerular filtration rate, *APTT* Activated partial thromboplastin time, *NT-proBNP* N-terminal pro-B-type natriuretic peptide, *hscTnI* Hypersensitive cardiac troponin I, *CK-MB* Creatine kinase isoenzymes, *HbA1c* Hemoglobin A1c, *ECMO* Extracorporeal membrane oxygenation, *ARDS* Acute respiratory distress syndrome

### Primary outcomes

In sight of mortality, 387 of 4,922 patients in the T2DM group performed better than 3,323 of 48,108 patients in the non-T2DM group (7.9% vs. 6.9%, *p* = 0.013). The severity ratio in the T2DM group performed higher than the non-T2DM group (23.4 vs. 32.2, *p* < 0.001). We used Kaplan–Meier curves to compare survival time and status. The mortality risk was significantly higher in the T2DM group than in the non-T2DM group (Fig. S1).

**Fig. 2:**
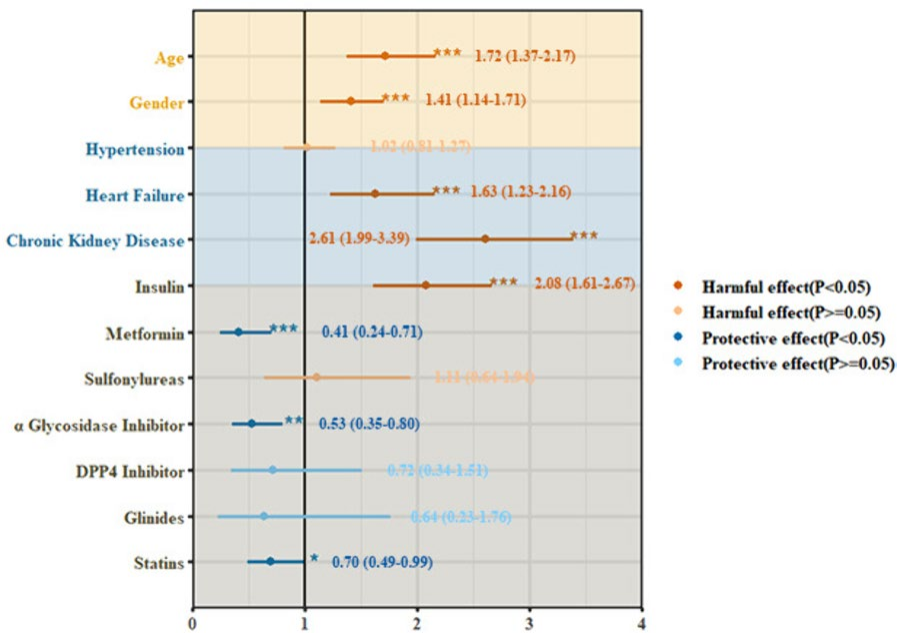
The forest plot to describe adjusted Hazard ratio for in-hospital all-cause mortality in the multivariate Cox regression model. The names of covariates included in the cox regression model were listed on the left of the figure and adjusted HR, 95% CI and *p*-value were shown in lines and points. (*p* < 0.1: *; *p* < 0.01: **; *p* < 0.001: ***.)

**Fig. 3:**
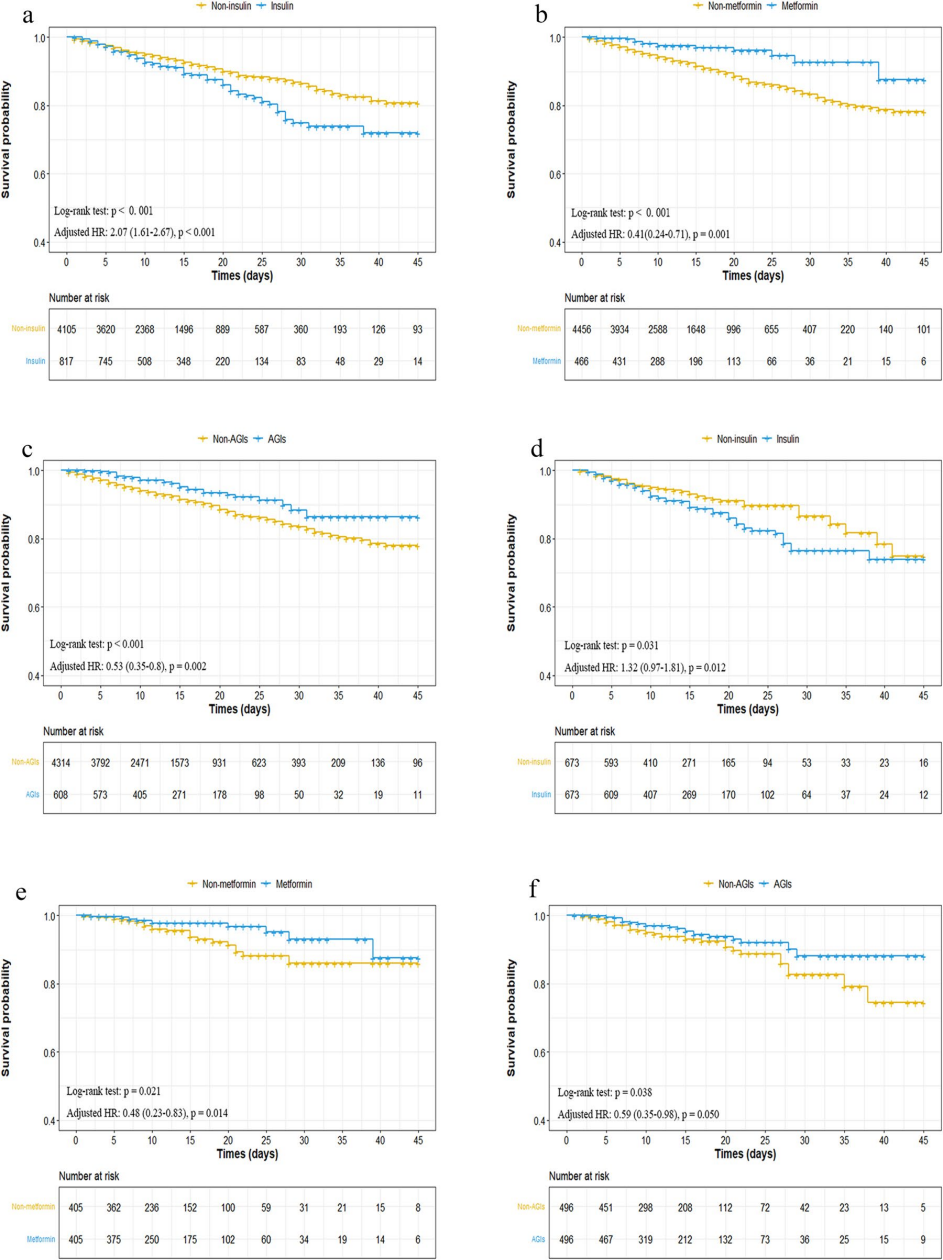
Effects of different treatments on in-hospital all-cause mortality for the patients with COVID-19 and T2DM. **a**, **b**, **c** The survival curves of in-hospital all-cause mortality for the patients with insulin, metformin or AGIs treatment before propensity score-matched analysis have been shown. **d**, **e**, **f** The survival curves of in-hospital all-cause mortality for the patients with insulin, metformin or AGIs treatment after propensity score-matched analysis have been shown

To clearly understand the effect of T2DM and drugs for T2DM and COVID-19, we chose metformin, insulin, sulfonylureas, glinides, α-glucosidase inhibitors (AGIs), and DPP4 inhibitors as our targets. After conducting univariate Cox regression, the HRs for all-cause mortality was 0.37 (95% CI, 0.22–0.61; *p* < 0.001) for metformin, 1.5 (95% CI, 1.2–1.9; *p* < 0.001) for insulin, 0.65 (95% CI, 0.39–1.10, *p* = 0.11) for sulfonylureas, 0.74 (95% CI, 0.37–1.50, *p* = 0.54) for glinides, 0.55 (95% CI, 0.38–0.79, *p* = 0.001) for AGIs, and 0.74 (95% CI, 0.37–1.50, *p* = 0.41) for DPP4 inhibitors. These variables were then included in the multivariate Cox regression model. After adjustment of age, sex, comorbidities, and inhospital medications, the use of insulin was associated with higher mortality (adjusted HR, 2.08; 95% CI, 1.61–2.67; *p* < 0.001; Fig. 2). In addition, the adjusted HRs for other variables were 0.41 (95% CI, 0.24–0.71; *p* = 0.001) for metformin, 1.11 (95% CI, 0.64–1.94; *p* = 0.702) for sulfonylureas, 0.64 (95% CI, 0.23–1.76, *p* = 0.384) for glinides, 0.72 (95% CI, 0.34–1.51, *p* = 0.382) for DPP4 inhibitors, and 0.53 (95% CI, 0.35–0.80, *p* = 0.002) for AGIs (Fig. 2). Kaplan–Meier curves showed that a higher risk of all-cause mortality in patients with COVID-19 and T2DM might be caused by the inhospital use of insulin; however, the use of metformin and AGIs performed a lower risk (*p* < 0.001), consistent with our above results (Fig. 3a–c and Fig. S2a–c).

Considering the existing imbalanced confounding factors, we conducted PSM. We matched 673 patients who were treated with insulin with who were treated without insulin, 405 patients who were treated with metformin with who were treated without metformin, and 496 patients who were treated with AGIs with who were treated without AGIs in a 1:1 ratio. In all, 1,346 patients were included in the PSM analysis. The balance was evaluated with SMD and *p*-values (Tables S1-S3). For the result of PSM, multivariate Cox regression performed consistent results with Kaplan-Meier curves which was also similar to the above (Fig. 3d–f). The adjusted HRs were 1.32 (95% CI, 1.03–1.81, *p* = 0.012) for insulin, 0.48 (95% CI, 0.23–0.83; *p* = 0.014) for metformin, and 0.59 (95% CI, 0.35–0.98; *p* = 0.050) for AGIs. PSM has also been applied to other drugs, such as sulfonylureas, glinides, and DPP4 inhibitors, at a ratio of 1:1. Tables S4-S6 shows their balance. Th0e multivariate Cox regression model and Kaplan-Meier curves revealed no statistically significant differences, similar to those before PSM (Fig. S2d-f).

According to the aforementioned results, we hypothesized the presence of a strong relation between insulin, metformin, and AGIs uses and the higher risk.

### Secondary outcomes

ARDS, invasive mechanical ventilation, acute kidney injury, and extracorporeal membrane oxygenation (ECMO) were chosen as secondary outcomes. Acute kidney injury appeared less than in the insulin group than in the non-insulin group (10.2% vs. 7.8%, *p* = 0.033), and the patients using invasive mechanical ventilation and ECMO were more in the insulin group than in the non-insulin group (invasive mechanical ventilation, 10.6% vs. 3.0%, *p* < 0.001; ECMO, 1.2% vs. 0.3%, *p* = 0.001; Table S1). However, the incidences of ARDS and acute kidney injury were significantly lower in the metformin group than in the non-metformin group (ARDS, 0.6% vs. 3.0%, *p* = 0.006; acute kidney injury, 3.2% vs. 8.8%, *p* < 0.001; Table S2). ARDS also occurred less in the AGI group (1.0% vs. 3.0%; *p* = 0.007; Table S3). Multivariate Cox regression analysis performed the adjusted HRs 1.25 (95% CI, 0.96–1.63; *p* = 0.100) for acute kidney injury, 4.12 (95% CI, 3.03–5.59, *p* < 0.001) for invasive mechanical ventilation, and 2.39 (95% CI, 0.89–6.42, *p* = 0.084) for ECMO. The adjusted HR for metformin was 0.28 (95% CI, 0.08–0.96, *p* = 0.043) for ARDS and 0.45 (95% CI, 0.26–0.77, *p* = 0.004) for acute kidney injury and the adjusted HR for AGIs associated with the risk of ARDS was 0.38 (95% CI, 0.15–0.94, *p* = 0.037; Table S7). Table S8 shows the adjusted HR for other drugs in the secondary outcomes. The results of the analysis of secondary outcomes suggested that the use of insulin was associated with a poor prognosis and that the use of metformin or AGIs was associated with a good prognosis.

### Comparison between insulin treatment and other antidiabetic drugs

**Fig. 4:**
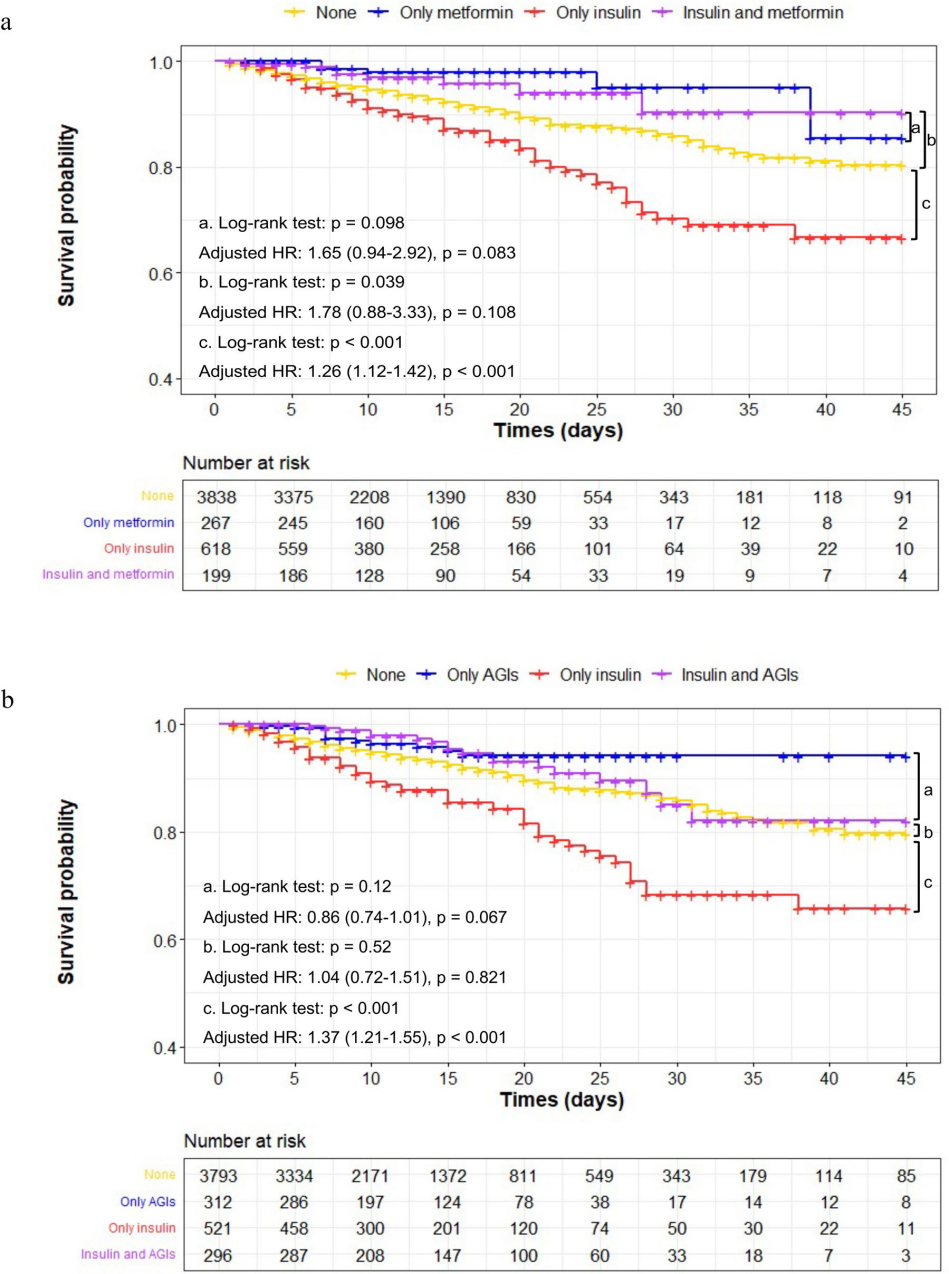
Joint analysis of insulin and metformin or AGIs treatment in patients with COVID-19 and T2DM. **a** Joint analysis of insulin and metformin treatment was conducted and shown by the survival curves of in-hospital all-cause mortality for patients with COVID-19 and T2DM; **b** Joint analysis of insulin and AGIs treatment was conducted and shown by the survival curves of in-hospital all-cause mortality for patients with COVID-19 and T2DM

The multivariate Cox regression model, Kaplan-Meier curves, and PSM revealed that insulin treatment was associated with a higher risk of all-cause mortality, while metformin and AGI treatments were associated with a lower risk. This contradicts our findings. To ensure accuracy and improve credibility, we selected patients taking the three drugs to compare the advantages and disadvantages of the combination of different drugs. Because of the opposing influence of insulin treatment, we first compared insulin and metformin treatments. The mortality of patients who received insulin alone was significantly higher than that of patients who received metformin alone (13.3% vs. 2.3%, *p* < 0.001). Kaplan-Meier curves showed that patients treated with metformin alone showed a significantly lower risk of all-cause mortality than those who received combined medication or insulin alone (Fig. 4a). The adjusted HR for individual insulin treatment was 1.26 (95%CI, 1.12–1.42, *p* < 0.001). Similar results were obtained in the comparison between insulin and AGI treatments. The mortality rates of patients who received the insulin or AGI treatment alone were 14.6% and 3.8%, respectively (*p* < 0.001). The adjusted HR for individual insulin treatment was 1.37 (95% CI, 1.21–1.55, *p* < 0.001; Fig. 4b). Insulin treatment is associated with a poor prognosis in patients with COVID-19 and diabetes.

### Subgroup analysis

**Fig. 5:**
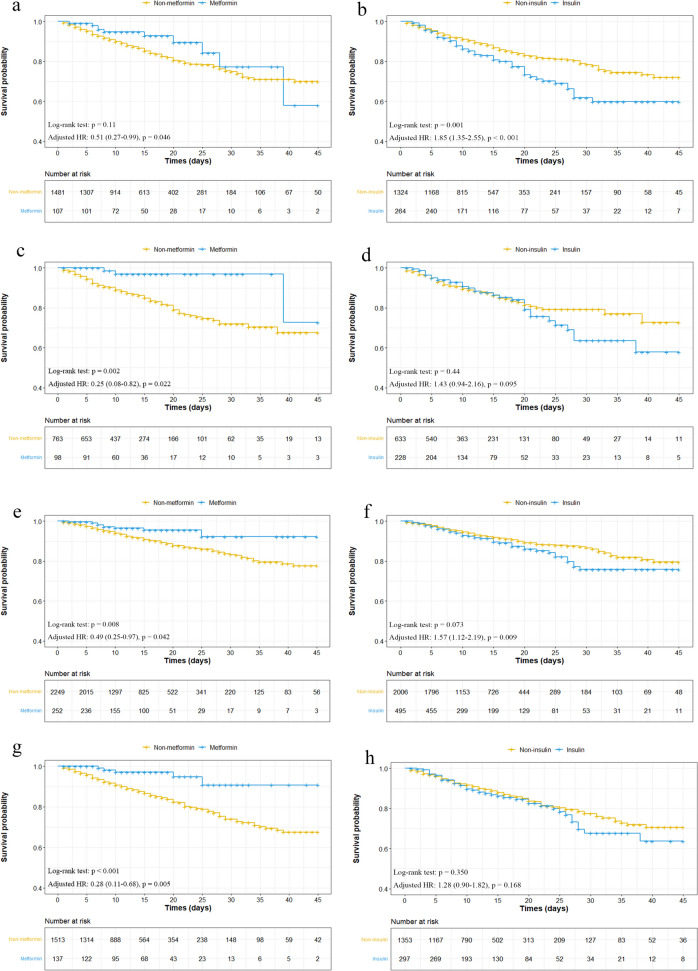
Subgroup analysis of different clinical conditions in patients with metformin or insulin treatment. **a**, **b** The survival curves of in-hospital mortality for critically ill patients with metformin or insulin treatment were shown; **c**, **d** The survival curves of in-hospital mortality for patients under poorly controlled glucose (glucose > 10 mmol/L) with metformin or insulin treatment were shown; **e**, **f** The survival curves of in-hospital mortality for patients under poorly controlled HbA1c (HbA1c > 6.5%) with metformin or insulin treatment were shown; **g**, **h** The survival curves of in-hospital mortality for patients under high NT-proBNP on admission (NT-proBNP > 265 mmol/L) with metformin or insulin treatment were shown

To observe the influence of antidiabetic drugs on mortality in different inhospital conditions and reduce the error from different laboratory results, we chose insulin and metformin use and compared their effects on all-cause mortality in different situations. First, we used Kaplan-Meier curves and multivariate Cox regression analyses in critically ill patients. Insulin treatment showed a higher risk of mortality (adjusted HR, 1.85, 95% CI, 1.35–2.55, *p* < 0.001), and metformin treatment showed a lower risk (adjusted HR, 0.51, 95% CI, 0.27–0.99, *p* = 0.046; Fig. 5a and b). Therefore, we hypothesized that different blood glucose levels might also have an influence. We chose fasting blood glucose and glycated hemoglobin (HbA1c) levels to compare treatments. The statistical analysis showed that in patients with poor glucose control and blood glucose levels > 10 mmol/L, insulin treatment was also associated with a higher risk, and metformin treatment was associated with a lower risk. The adjusted HRs were 0.25 (95% CI, 0.08–0.82, *p* = 0.022) for metformin treatment and 1.43 (95% CI, 0.94–2.16, *p* = 0.095) for insulin treatment (Fig. 5c and d). In the HbA1c > 6.5% group the adjusted HR was 0.49 (95%CI, 0.25–0.97, *p* = 0.042) for metformin treatment and 1.57 (95% CI,1.12–2.19, *p* = 0.009) for insulin treatment (Fig. 5e–f). Finally, we analyzed them in patients with heart injury whose NT-proBNP level exceeds 265 ng/µL. We obtained similar results. The adjusted HR was 0.28 (95% CI, 0.11–0.68, *p* = 0.005) for metformin treatment and 1.28 (95% CI, 0.90–1.82, *p* = 0.168) for insulin treatment (Fig. 5g and h). Fig. S3a-f shows patients with a blood glucose level ≤ 10 mmol/L, HbA1c ≤ 6.5%, or NT-proBNP < 265 ng/µL, with the same results as shown above.

In short, patients classified into different groups showed similar outcomes. Insulin treatment was always associated with decreased survival, while metformin treatment was associated with increased survival.

### Dynamic profiles

**Fig. 6:**
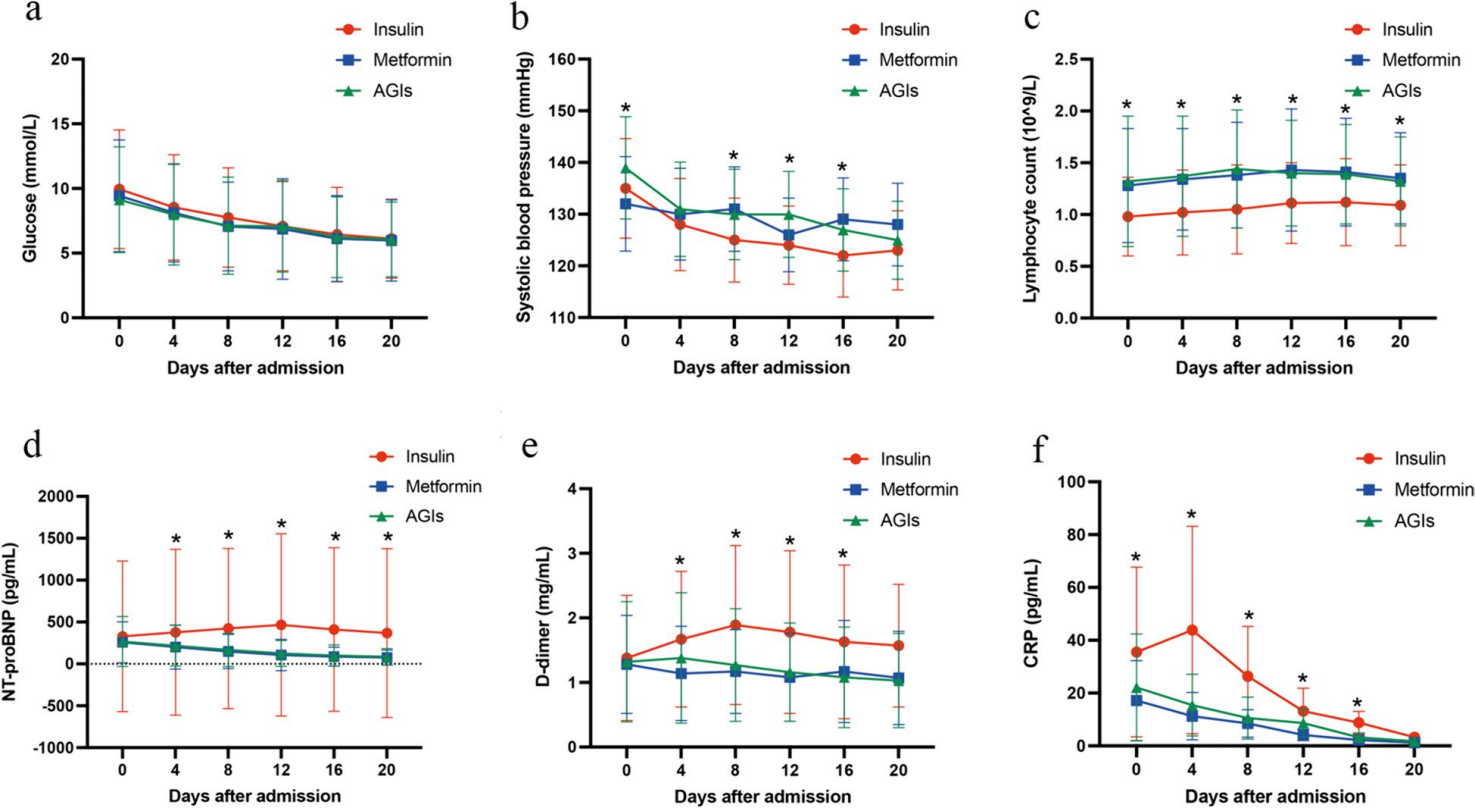
Dynamic Profile of Vital Signs and Laboratory Parameters in All Patients with COVID-19 and T2D with insulin, metformin and AGIs treatment. **a**, **b**, **c**, **d**, **e**, **f** The line charts showed dynamic change of blood glucose (mmol/L), systolic blood pressure (mmhg), lymphocyte count (10^9/L), NT-proBNP (pg/ml), D-dimer (mg/ml) and C-reaction protein (pg/ml) with days after admission

To evaluate the characteristics and dynamic changes after treatment, we monitored the vital signs and laboratory results, including blood glucose, systolic blood pressure, lymphocyte count, NT-proBNP level, D-dimer level, and C-reactive protein (CRP) level. Blood glucose levels in all three groups became lower after admission and tended to stabilize (Fig. 6a). Contrary to the metformin and AGI groups, the insulin group showed lower systolic blood pressure and lymphocyte counts and higher NT-proBNP and D-dimer levels (Fig. 6b–e). This suggests the possibility of an association among insulin treatment, heart injury, and coagulation disorders. The CRP level reached its highest on day 4 in the insulin group and became gradually lower but still remained higher than that in the other groups, indicating that patients in the insulin group might develop more severe infection and inflammation than those in the other groups (Fig. 6f). These results suggested that insulin treatment may worsen organ injury during infection, leading to increased mortality.

## Discussion

In the present retrospective study, insulin and other antidiabetic drug were investigated and significant statistical differences in all-cause mortality in patients treated with insulin, metformin, and AGIs were shown. Treatments with metformin and AGIs were associated with a lower mortality risk, while insulin treatment might bring about increased mortality. A similar result was observed in the subgroup analysis, classified according to baseline characteristics. These results suggest that insulin treatment came to worse outcomes, whereas metformin and AGIs were associated with better outcomes.

Evidence suggests that preexisting T2DM is significantly associated with the morbidity and mortality of COVID-19 [15–17]. Approximately 20% of patients with COVID-19 develop T2DM, and 10% of patients are diagnosed with T2DM [18, 19]. Compared to patients without T2DM, those with T2DM may have a higher ratio of critical illness, mortality, and severe complications, such as respiratory failure and heart failure [17, 20–22]. Poor disease control in patients with T2DM may promote severe infections, including those by SARS-CoV-1, H1N1, and Middle East respiratory syndrome coronavirus [23, 24]. High blood glucose levels may increase angiotensin-converting enzyme 2 levels in lung tissue, which mediates the SARS-CoV-2 infection into cells and aggravate infection [25, 26]. Meanwhile, a study found that severe infection could also worsen the hyperglycemic state, creating a vicious circle [2, 27]. Therefore, the management of T2DM in patients with COVID-19 is worthy of attention.

No consensus exists regarding which antidiabetic drug should be prioritized for treating patients with COVID-19 and T2DM. Some observational and retrospective studies suggested that metformin treatment might exert a favorable effect, while others suggested that metformin treatment might result in poor outcomes, such as acidosis and severe respiratory difficulty [10, 28]. In some in vitro studies, metformin acted against SARS-CoV-2 with anti-inflammation actions, including reducing interleukin-1β and interleukin-6 levels and inflammasome activation [29–32]. A randomized controlled trial published in the *NEJM* provided evidence that metformin did not prevent the occurrence of hypoxemia, hospitalization, or death from COVID-19 [33]. AGIs might disrupt SARS-CoV-2 replication, similar to SARS-CoV-1 [34–36]. In an in vitro study by Miglustat and Celgosivir, two AGIs showed antiviral potential against SARS-CoV-2, particularly against severe respiratory infection [37]. A clinical study on the positive effect of AGIs showed that AGI treatment performed lower mortality [38].

The role of insulin in patients with COVID-19 and T2DM remains controversial. In early stages, most statements and guidelines recommended insulin injections to control hyperglycemia [15, 39]. However, our previous laboratory study found that insulin treatment might significantly worsen viral infections by elevating interleukin-6 levels and the ratio of severe complications to death [14]. Randomized controlled trials of insulin remain lacking, and more evidence is required for clinical practice.

In the present study, metformin and AGI treatments were associated with a lower risk of mortality, whereas the insulin treatment was associated with increased mortality. Clinical practitioners should be cautious when administering insulin to patients with COVID-19; metformin or AGIs may be better choices.

This study still has several limitations. First, the data were collected from December 2019 to August 2021, which may have lagged behind the current pandemic situation. Due to the urgent state at that time, comprehensive monitoring of various indices was lacking in inpatients. Second, this was only retrospective that could not describe causal effects and only provided clinical evidence for therapy. Therefore, more randomized clinical trials are required in the future.

## Conclusion

Insulin was associated with a higher risk of all-cause mortality in patients with COVID-19 with T2DM, while metformin and AGIs were associated with a lower risk. In patients with COVID-19 and T2DM, metformin or AGIs might be prioritized over insulin.

## Materials and methods

### Study design

This study included patients with COVID-19 from 138 hospitals in the Hubei Province. They were confirmed COVID-19 on the basis of the China and World Health Organization guidelines, and those who died or were discharged between December 29, 2019 (i.e., when the first patients were admitted) and August 31, 2021, were included in the present study. Those aged < 18 years and lacking important basic information, laboratory test results for SARS-CoV-2 were excluded. The electronic medical record database provided all data from the Health Commission of Hubei Province.

To deal with center effects, firstly, we have excluded any hospitals with less than 100 cases. Then, in the implementation phase, we used the same electronic management system to collect data quickly and correctly and the same diagnosis and treatment criteria for COVID-19 which was published by Hubei Provincial Health Commission. Meantime all medical workers have been professionally trained to collect and process data in the uniform standard.

Finally, 53,030 inpatients with COVID-19 were enrolled in this study. Of them, 4,922 patients suffered from T2DM diagnosed according to the guidelines published by the Chinese Diabetes Society and were managed according the recommendation of the Chinese Diabetes Society [40]. 

### Data collection and endpoint definitions

The examination of data from electronic medical records were conducted individually by three researchers. The information included age, sex, comorbidities, vital signs, laboratory tests, therapeutic strategy, and outcomes.

Primary endpoint was defined as all-cause death and second outcomes were defined as life-threatening acute injury and need for life-support treatment such as ARDS, invasive mechanical ventilation, acute kidney injury and extracorporeal membrane oxygenation (ECMO).

### Propensity score-matching (PSM) and Cox regression analysis

To balance the differences in covariates between groups, PSM was performed. We adjusted for confounding factors including sex; age; severity; laboratory results, such as white blood cell count, glutamic-pyruvic transaminase level, creatinine level, N-terminal pro–B-type natriuretic peptide (NT-proBNP) level and other associated diseases, such as chronic kidney diseases and heart failure. These indicators were selected referred to published risk factors and these included in other PSM studies [3, 4, 14, 28]. Statistical analyses were performed by R MatchIt Package. The caliper width was set as 0.05. The standardized mean difference (SMD) was used to evaluate the balance of covariates after matching and *p*-value < 0.1 indicated successful matching.

Multivariate Cox regression models were used to further adjust imbalanced covariates. Results are expressed as hazard ratio (HR) and 95% confidence interval (CI). Survival analysis methods were used to compare the timing of endpoint events among subgroups.

### Statistical analysis

Statistical analyses were performed using SPSS (version 24.0; IBM, Armonk, NY, USA) and R (version 4.1.1; R Foundation for Statistics Computing, Vienna, Austria). D’Agostino’s and Pearson’s omnibus normality tests were used to assess the distribution and homoscedasticity of each dataset. Continuous normally distributed data were expressed as mean (standard deviation) and compared using the Student’s *t*-test. Median (interquartile range) were used to express continuous non-normally distributed data. They were compared using the Wilcoxon rank-sum test. Categorical data are expressed as n (percentage) and compared by the chi-square, Fisher’s exact, and Cochran–Mantel–Haenszel tests, as appropriate.

### Patient and public involvement

In our study it was not appropriate or possible to involve patients or the public in the design, or conduct, or reporting, or dissemination plans of our research because any their identifiable information such as ID and telephone were hid by the Hubei Provincial Health Commission and patients may be lack of related professional knowledge.

### Supplementary Information

**Supplementary Material 1.**

## Data Availability

The data underlying this article will be shared on reasonable request to the corresponding author.

## References

[1] Katulanda P, Dissanayake HA, Ranathunga I, Ratnasamy V, Wijewickrama PSA, Yogendranathan N (2020). Prevention and management of COVID-19 among patients with diabetes: an appraisal of the literature. Diabetologia.

[2] Zhu L, She ZG, Cheng X, Qin JJ, Zhang XJ, Cai J (2020). Association of blood glucose control and outcomes in patients with COVID-19 and pre-existing type 2 diabetes. Cell Metab.

[3] Williamson EJ, Walker AJ, Bhaskaran K, Bacon S, Bates C, Morton CE (2020). Factors associated with COVID-19-related death using OpenSAFELY. Nature.

[4] Huang C, Wang Y, Li X, Ren L, Zhao J, Hu Y (2020). Clinical features of patients infected with 2019 novel coronavirus in Wuhan, China. Lancet.

[5] Gianchandani R, Esfandiari NH, Ang L, Iyengar J, Knotts S, Choksi P (2020). Managing hyperglycemia in the COVID-19 inflammatory storm. Diabetes.

[6] Jolobe OMP (2022). Post-COVID-19 diabetes in the context of long COVID. Am J Emerg Med.

[7] Kunal S, Madan M, Tarke C, Gautam DK, Kinkar JS, Gupta K (2022). Emerging spectrum of post-COVID-19 syndrome. Postgrad Med J.

[8] Gupta R, Ghosh A, Singh AK, Misra A (2020). Clinical considerations for patients with diabetes in times of COVID-19 epidemic. Diabetes Metab Syndr.

[9] Longo M, Caruso P, Maiorino MI, Bellastella G, Giugliano D, Esposito K (2020). Treating type 2 diabetes in COVID-19 patients: the potential benefits of injective therapies. Cardiovasc Diabetol.

[10] Chan LE, Casiraghi E, Laraway B, Coleman B, Blau H, Zaman A (2022). Metformin is associated with reduced COVID-19 severity in patients with prediabetes. Diabetes Res Clin Pract.

[11] Bailey CJ, Gwilt M (2022). Diabetes, metformin and the clinical course of Covid-19: outcomes, mechanisms and suggestions on the therapeutic use of metformin. Front Pharmacol.

[12] Bramante CT, Buse J, Tamaritz L, Palacio A, Cohen K, Vojta D (2021). Outpatient metformin use is associated with reduced severity of COVID-19 disease in adults with overweight or obesity. J Med Virol.

[13] Ghany R, Palacio A, Dawkins E, Chen G, McCarter D, Forbes E (2021). Metformin is associated with lower hospitalizations, mortality and severe coronavirus infection among elderly medicare minority patients in 8 states in USA. Diabetes Metab Syndr.

[14] Yu B, Li C, Sun Y, Wang DW (2021). Insulin treatment is associated with increased mortality in patients with COVID-19 and type 2 diabetes. Cell Metab.

[15] Bornstein SR, Rubino F, Khunti K, Mingrone G, Hopkins D, Birkenfeld AL (2020). Practical recommendations for the management of diabetes in patients with COVID-19. Lancet Diabetes Endocrinol.

[16] McGurnaghan SJ, Weir A, Bishop J, Kennedy S, Blackbourn LAK, McAllister DA (2021). Risks of and risk factors for COVID-19 disease in people with diabetes: a cohort study of the total population of Scotland. Lancet Diabetes Endocrinol.

[17] Zhang Y, Luo W, Li Q, Wang X, Chen J, Song Q (2022). Risk factors for death among the First 80 543 coronavirus disease 2019 (COVID-19) cases in China: relationships between age, underlying disease, case severity, and region. Clin Infect Dis.

[18] Zhou F, Yu T, Du R, Fan G, Liu Y, Liu Z (2020). Clinical course and risk factors for mortality of adult inpatients with COVID-19 in Wuhan, China: a retrospective cohort study. Lancet.

[19] Guo L, Shi Z, Zhang Y, Wang C, Do Vale Moreira NC, Zuo H (2020). Comorbid diabetes and the risk of disease severity or death among 8807 COVID-19 patients in China: a meta-analysis. Diabetes Res Clin Pract.

[20] Llauradó G, Vlacho B, Wargny M, Ruan Y, Franch-Nadal J, Domingo P (2022). The association between macrovascular complications and intensive care admission, invasive mechanical ventilation, and mortality in people with diabetes hospitalized for coronavirus disease-2019 (COVID-19). Cardiovasc Diabetol.

[21] Wang D, Hu B, Hu C, Zhu F, Liu X, Zhang J (2020). Clinical characteristics of 138 hospitalized patients with 2019 novel coronavirus-infected pneumonia in Wuhan, China. JAMA.

[22] The epidemiological characteristics of an outbreak of 2019 novel coronavirus diseases (COVID-19) in China. Zhonghua Liu Xing Bing Xue Za Zhi. 2020;41(2):145−51. 10.3760/cma.j.issn.0254-6450.2020.02.003. 10.3760/cma.j.issn.0254-6450.2020.02.003 32064853

[23] Badawi A, Ryoo SG (2016). Prevalence of diabetes in the 2009 Influenza A (H1N1) and the Middle East respiratory syndrome coronavirus: a systematic review and Meta-analysis. J Public Health Res.

[24] Yang JK, Feng Y, Yuan MY, Yuan SY, Fu HJ, Wu BY (2006). Plasma glucose levels and diabetes are independent predictors for mortality and morbidity in patients with SARS. Diabet Med.

[25] Hoffmann M, Kleine-Weber H, Schroeder S, Krüger N, Herrler T, Erichsen S (2020). SARS-CoV-2 cell entry depends on ACE2 and TMPRSS2 and is blocked by a clinically proven protease inhibitor. Cell.

[26] Roca-Ho H, Riera M, Palau V, Pascual J, Soler MJ (2017). Characterization of ACE and ACE2 expression within different organs of the NOD mouse. Int J Mol Sci.

[27] Singh AK, Khunti K (2022). COVID-19 and diabetes. Annu Rev Med.

[28] Cheng X, Liu YM, Li H, Zhang X, Lei F, Qin JJ (2020). Metformin is Associated with higher incidence of Acidosis, but not mortality, in individuals with COVID-19 and pre-existing type 2 diabetes. Cell Metab.

[29] Gordon DE, Jang GM, Bouhaddou M, Xu J, Obernier K, White KM (2020). A SARS-CoV-2 protein interaction map reveals targets for drug repurposing. Nature.

[30] Schaller MA, Sharma Y, Dupee Z, Nguyen D, Urueña J, Smolchek R, et al. Ex vivo SARS-CoV-2 infection of human lung reveals heterogeneous host defense and therapeutic responses. JCI Insight. 2021;6. 10.1172/jci.insight.148003. 10.1172/jci.insight.148003 PMC8492301 34357881

[31] Postler TS, Peng V, Bhatt DM, Ghosh S (2021). Metformin selectively dampens the acute inflammatory response through an AMPK-dependent mechanism. Sci Rep.

[32] Xian H, Liu Y, Rundberg Nilsson A, Gatchalian R, Crother TR, Tourtellotte WG (2021). Metformin inhibition of mitochondrial ATP and DNA synthesis abrogates NLRP3 inflammasome activation and pulmonary inflammation. Immunity.

[33] Bramante CT, Huling JD, Tignanelli CJ, Buse JB, Liebovitz DM, Nicklas JM (2022). Randomized trial of metformin, ivermectin, and fluvoxamine for Covid-19. N Engl J Med.

[34] Ritchie G, Harvey DJ, Feldmann F, Stroeher U, Feldmann H, Royle L (2010). Identification of N-linked carbohydrates from severe acute respiratory syndrome (SARS) spike glycoprotein. Virology.

[35] Fukushi M, Yoshinaka Y, Matsuoka Y, Hatakeyama S, Ishizaka Y, Kirikae T (2012). Monitoring of S protein maturation in the endoplasmic reticulum by calnexin is important for the infectivity of severe acute respiratory syndrome coronavirus. J Virol.

[36] Williams SJ, Goddard-Borger ED (2020). α-glucosidase inhibitors as host-directed antiviral agents with potential for the treatment of COVID-19. Biochem Soc Trans.

[37] Rajasekharan S, Milan Bonotto R, Nascimento Alves L, Kazungu Y, Poggianella M, Martinez-Orellana P (2021). Inhibitors of protein glycosylation are active against the coronavirus severe acute respiratory syndrome coronavirus SARS-CoV-2. Viruses.

[38] Li J, Wei Q, McCowen KC, Xiong W, Liu J, Jiang W (2022). Inpatient use of metformin and acarbose is associated with reduced mortality of COVID-19 patients with type 2 diabetes mellitus. Endocrinol Diabetes Metab.

[39] Sardu C, D’Onofrio N, Balestrieri ML, Barbieri M, Rizzo MR, Messina V (2020). Outcomes in patients with hyperglycemia affected by COVID-19: can we do more on glycemic control?. Diabetes Care.

[40] Jia W, Weng J, Zhu D, Ji L, Lu J, Zhou Z (2019). Standards of medical care for type 2 diabetes in China 2019. Diabetes Metab Res Rev.

