# Supplementary material for: Effects of different treatments for type 2 diabetes mellitus on mortality of coronavirus disease from 2019 to 2021 in China: a multi-institutional retrospective study
Source: Mol Biomed. 2024 May 17;5:18. doi: 10.1186/s43556-024-00183-1 (PMC11099001; doi:10.1186/s43556-024-00183-1)
Supplement: Supplementary file 1 — Supplementary Material 1. [file 43556_2024_183_MOESM1_ESM.docx]

**Effects of Different Treatments for Type 2 Diabetes Mellitus on Mortality of Coronavirus Disease from 2019 to 2021 in China: A Multi-institutional Retrospective Study**

Ke Xu, MD, Wu He, MD, Bo Yu^1^, MD, Kaineng Zhong^2^, MD, Da Zhou^2^, MD, Dao Wen Wang^1*^ MD, PhD

^1^Division of Cardiology, Department of Internal Medicine, Tongji Hospital, Tongji Medical College, Huazhong University of Science and Technology; Hubei Key Laboratory of Genetics and Molecular Mechanisms of Cardiological Disorders, Wuhan 430030, China; ^2^Hubei Provincial Health Commission, Wuhan 430079, China

Corresponding author:

Dao Wen Wang

Division of Cardiology, Department of Internal Medicine

Tongji Hospital, Tongji Medical College

Huazhong University of Science & Technology

1095# Jiefang Ave.

Wuhan 430030, China

Email: [dwwang@tjh.tjmu.edu.cn](mailto:dwwang@tjh.tjmu.edu.cn)

**Supplementary Figures**

**Figure S1.** **The Kaplan–Meier survival curve of all in-hospital mortality for COVID-19 patients with and without T2D.**

**Figure S2. Effects of different treatments on in-hospital all-cause mortality for the patients with COVID-19 and T2DM.**

**A, B, C** The Kaplan–Meier survival curves of in-hospital all-cause mortality for the patients with sulfonylureas, glinides or DPP4 treatment before propensity score-matched analysis. **D, E, F** The Kaplan–Meier survival curves of in-hospital all-cause mortality for the patients with sulfonylureas, glinides or DPP4 treatment after propensity score-matched analysis.

**Figure S3. Other three subgroups analysis of different clinical conditions in patients with metformin or insulin treatment**

**A, B** The Kaplan–Meier survival curves of in-hospital mortality for patients under well-controlled glucose (glucose <= 10 mmol/L) with metformin or insulin treatment; **C, D** The Kaplan–Meier survival curves of in-hospital mortality for patients under well-controlled HbA1c (HbA1c <= 6.5%) with metformin or insulin treatment; **E, F** The Kaplan–Meier survival curves of in-hospital mortality for patients under normal NT-proBNP on admission (NT-proBNP <= 265 mmol/L) with metformin or insulin treatment.

**Supplementary Figure S1**


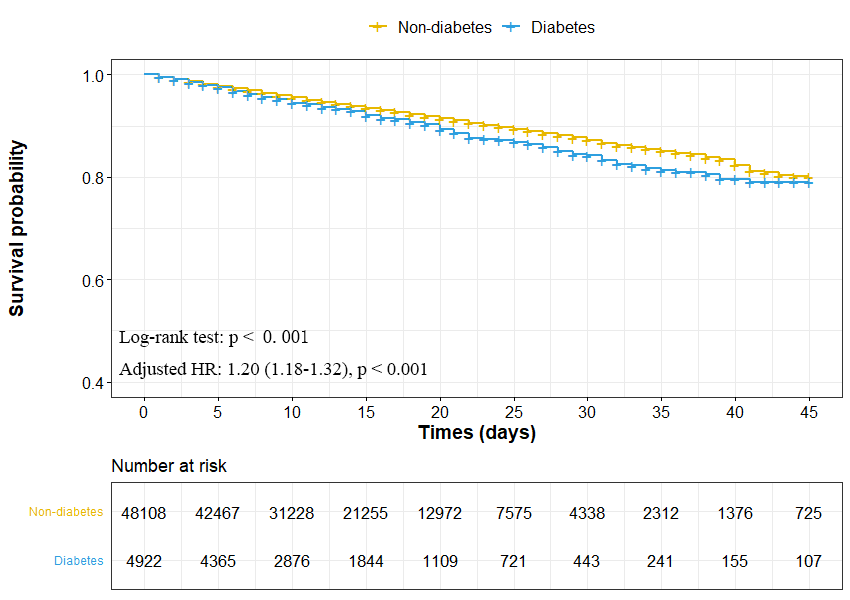


**Supplementary Figure S2**


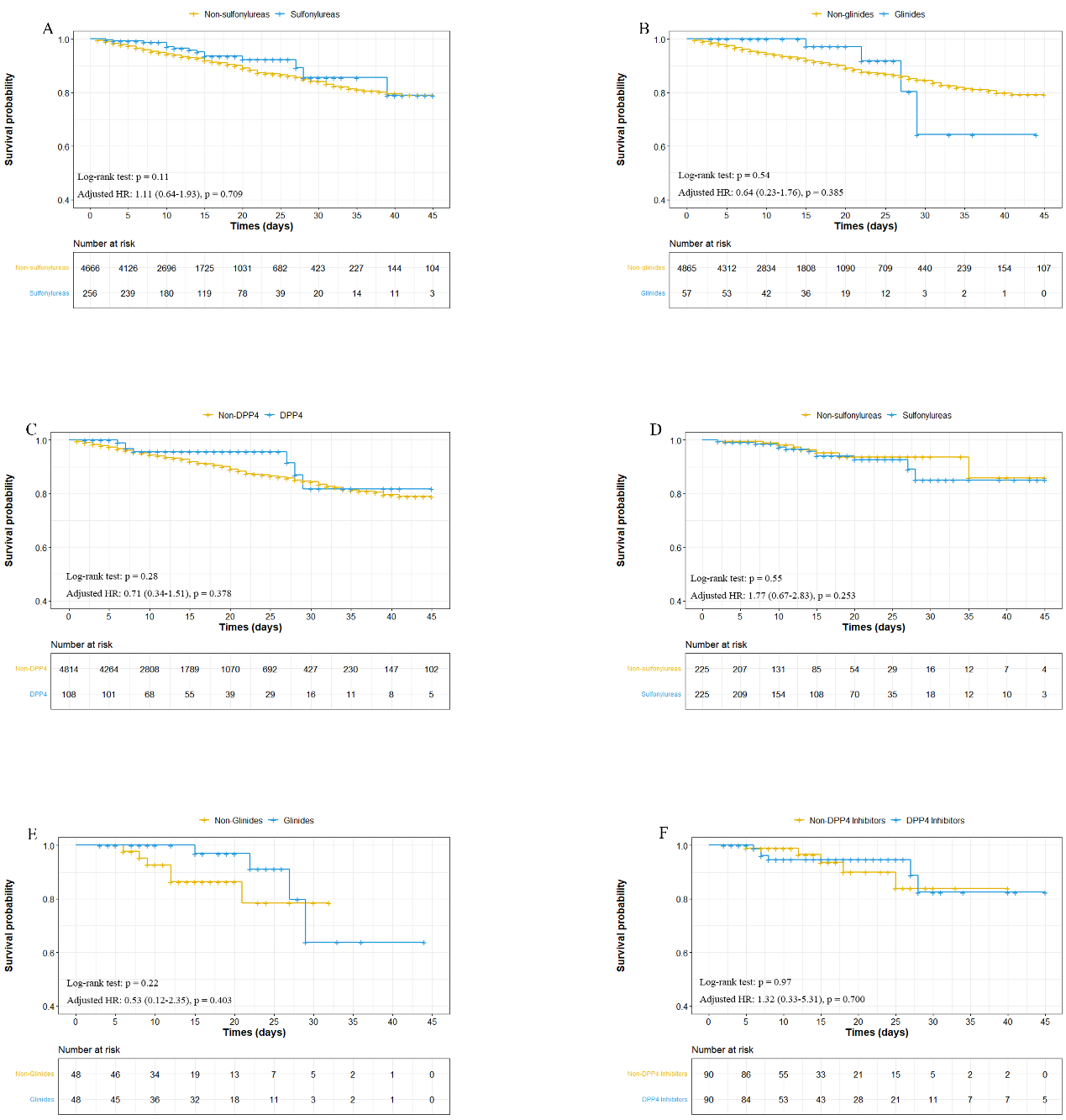


**Supplementary Figure S3**


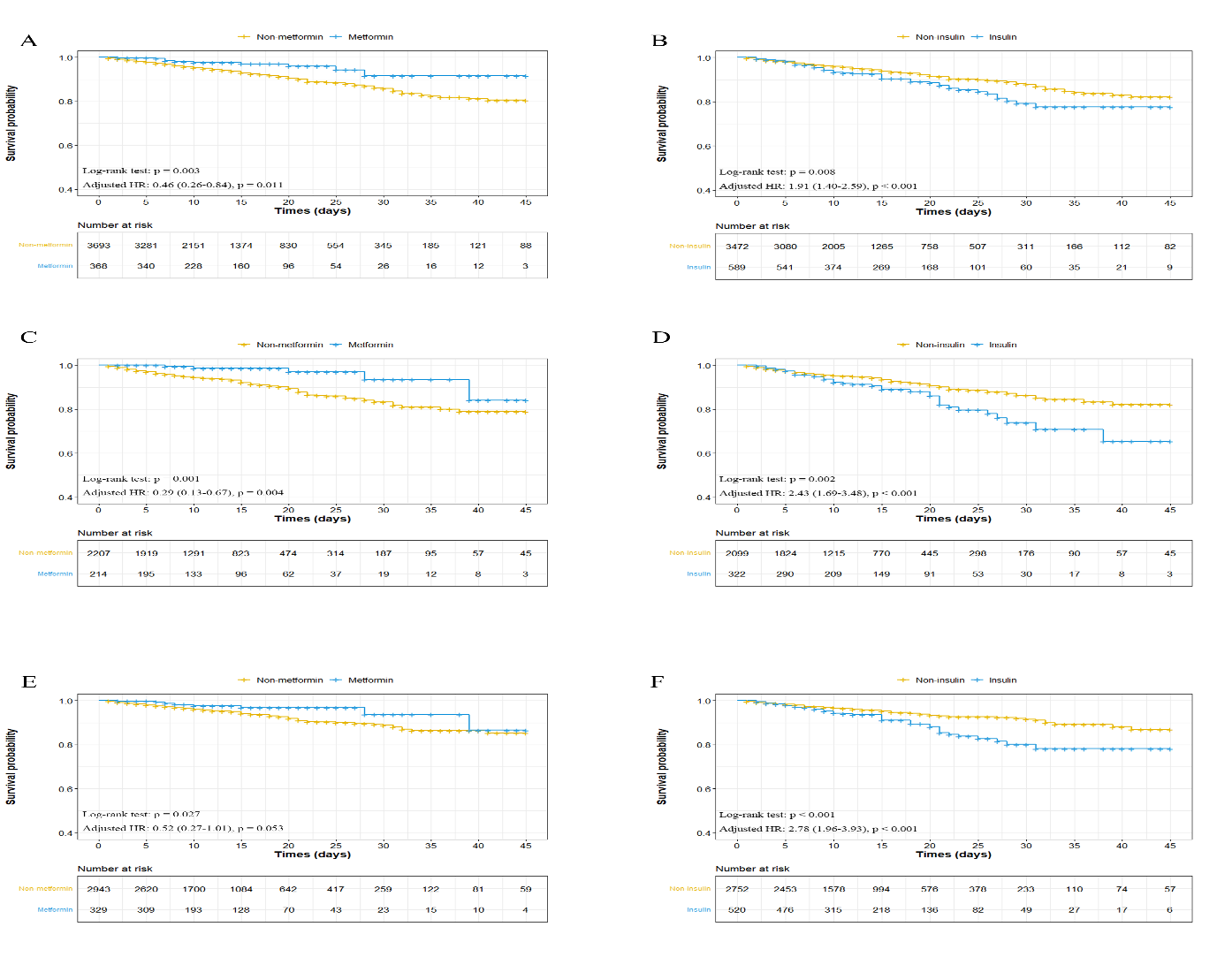


**Supplementary Table S1: Characteristics of Patients with COVID-19 and the Type 2 Diabetes in Insulin and Non-Insulin groups Before and After PSM**

|  | **All patients with diabetes (n=4922)** | | | **PSM 1:1 (n=1346)** | | |
| --- | --- | --- | --- | --- | --- | --- |
|  | **Patients with insulin (n=817)** | **Patients without insulin (n= 4105)** | **P-value** | **Patients with insulin (n=673)** | **Patients without insulin (n=673)** | **P-value** |
| Gender: Male (%) | 450 (55.1) | 2180 (53.1) | 0.32 | 372 (54.7) | 382 (56.8) | 0.017 |
| Age, years | 67.00 [58.00, 74.00] | 66.00 [58.00, 76.00] | 0.586 | 67.00 [57.00, 74.00] | 66.00 [57.00, 74.00] | 0.653 |
| Age ≥65 years (%) | 478 (58.5) | 2328 (56.7) | 0.364 | 395 (58.7) | 361 (53.6) | 0.07 |
| Age＜65 years (%) | 339 (41.5) | 1777 (43.3) | 0.364 | 278 (41.3) | 312 (46.4) | 0.07 |
| Hospitalization time (days) | 13.00 [7.00, 20.00] | 11.00 [7.00, 18.00] | <0.001 | 12.00 [7.00, 20.00] | 12.00 [7.00, 19.00] | 0.68 |
| COVID-19 Severity (n %) |  |  |  |  |  |  |
| Mild | 310 (37.9) | 1512 (36.8) | 0.212 | 254 (37.7) | 234 (34.8) | 0.132 |
| Normal | 243 (29.7) | 1269 (30.9) |  | 206 (30.6) | 231 (34.3) |  |
| Severe | 195 (23.9) | 1051 (25.6) |  | 155 (23.0) | 167 (24.8) |  |
| Critical | 69 (8.4) | 273 (6.7) |  | 58 (8.6) | 41 (6.1) |  |
| **Vital signs** |  |  |  |  |  |  |
| Systolic blood pressure (mmHg) | 128.00 [120.00, 140.00] | 127.00 [114.00, 140.00] | 0.003 | 127.00 [119.00, 140.00] | 129.00 [120.00, 140.00] | 0.216 |
| Diastolic blood pressure (mmHg) | 78.00 [70.00, 87.00] | 80.00 [70.00, 90.00] | <0.001 | 77.00 [70.00, 87.00] | 77.00 [70.00, 85.00] | 0.973 |
| Respiratory rate (times per minute) | 20.00 [19.00, 20.00] | 20.00 [19.00, 20.00] | 0.245 | 20.00 [19.00, 20.00] | 20.00 [19.00, 20.00] | 0.6 |
| Pulse rate (times per minute) | 80.00 [74.00, 90.00] | 80.00 [74.00, 88.00] | 0.592 | 80.00 [74.00, 90.00] | 80.00 [74.00, 88.00] | 0.887 |
| Temperature (°C) | 36.50 [36.30, 36.70] | 36.50 [36.30, 36.70] | 0.002 | 36.50 [36.30, 36.70] | 36.50 [36.30, 36.70] | 0.84 |
| **Origin Comorbidities** |  |  |  |  |  |  |
| Hypertension (%) | 491 (60.1) | 2585 (63.0) | 0.131 | 398 (59.1) | 387 (57.5) | 0.580 |
| Hypercholesterolemia (%) | 419 (51.3) | 1921 (46.8) | 0.090 | 333 (49.5) | 330 (49.0) | 0.913 |
| Coronary heart disease (%) | 168 (20.6) | 1073 (26.1) | 0.001 | 138 (20.5) | 134 (19.9) | 0.839 |
| COPD (%) | 29 (3.5) | 238 (5.8) | 0.012 | 27 (4.0) | 42 (6.2) | 0.084 |
| Heart failure (%) | 95 (11.6) | 369 (9.0) | 0.022 | 72 (10.7) | 74 (11.0) | 0.930 |
| Arrhythmia (%) | 67 (8.2) | 317 (7.7) | 0.693 | 57 (8.5) | 59 (8.8) | 0.923 |
| CKD (%) | 83 (10.2) | 309 (7.5) | 0.014 | 63 (9.4) | 60 (8.9) | 0.850 |
| **Laboratory results** |  |  |  |  |  |  |
| **Routine blood test** |  |  |  |  |  |  |
| Red blood cell count, ×10^12^/L | 4.09 [3.66, 4.51] | 4.14 [3.70, 4.56] | 0.066 | 4.10 [3.68, 4.55] | 4.15 [3.72, 4.60] | 0.216 |
| White cell count, ×10^9^/L | 6.23 [4.97, 7.90] | 6.12 [4.81, 7.89] | 0.494 | 6.17 [4.95, 7.90] | 6.32 [5.03, 8.16] | 0.373 |
| Hemoglobin g/L | 123.00 [111.00, 136.00] | 125.00 [112.00, 137.00] | 0.053 | 123.00 [112.00, 137.00] | 125.00 [112.00, 138.00] | 0.242 |
| Neutrophil count, ×10^9^/L | 4.11 [3.05, 5.72] | 3.94 [2.90, 5.59] | 0.013 | 4.03 [3.04, 5.59] | 4.03 [3.07, 5.82] | 0.976 |
| Monocyte count, ×10^9^/L | 0.41 [0.31, 0.55] | 0.41 [0.31, 0.55] | 0.460 | 0.41 [0.31, 0.55] | 0.42 [0.31, 0.55] | 0.747 |
| Platelet count, ×10^9^/L | 189.00 [150.00, 234.00] | 195.00 [156.00, 237.00] | 0.033 | 189.00 [149.00, 234.00] | 196.00 [159.00, 235.00] | 0.118 |
| **Blood biochemistry** |  |  |  |  |  |  |
| Alanine aminotransferase, U/L | 19.70 [14.00, 32.00] | 20.60 [14.00, 32.60] | 0.290 | 20.00 [13.80, 33.00] | 22.00 [14.00, 35.30] | 0.12 |
| Aspartate aminotransferase, U/L | 21.00 [15.40, 31.00] | 21.90 [16.70, 30.70] | 0.056 | 21.00 [15.30, 31.00] | 21.50 [16.10, 29.80] | 0.289 |
| Lactate dehydrogenase, U/L | 190.00 [154.80, 253.00] | 186.70 [152.40, 245.00] | 0.144 | 187.00 [153.00, 246.00] | 188.00 [154.00, 252.00] | 0.726 |
| Total bilirubin, umol/L | 10.60 [8.20, 14.70] | 10.60 [7.90, 14.50] | 0.595 | 10.63 [8.20, 14.70] | 10.70 [7.90, 15.00] | 0.896 |
| Total protein, g/L | 67.10 [62.00, 71.70] | 67.20 [61.90, 72.10] | 0.423 | 67.20 [62.20, 71.70] | 67.20 [62.10, 72.40] | 0.471 |
| Globulin, g/L | 28.30 [24.60, 32.10] | 27.90 [24.60, 31.60] | 0.082 | 28.20 [24.50, 32.10] | 28.10 [24.90, 31.80] | 0.793 |
| Albumin, g/L | 38.40 [34.30, 41.80] | 39.00 [34.60, 43.10] | 0.001 | 38.50 [34.50, 42.10] | 38.70 [34.50, 42.80] | 0.28 |
| Alkaline phosphatase, U/L | 69.00 [55.00, 87.00] | 72.00 [58.00, 91.00] | 0.002 | 70.00 [56.00, 87.00] | 70.00 [55.80, 90.00] | 0.427 |
| Total cholesterol, mmol/L | 4.08 [3.44, 4.84] | 4.14 [3.45, 4.96] | 0.355 | 4.13 [3.47, 4.88] | 4.12 [3.43, 4.90] | 0.757 |
| Triglyceride, mmol/L | 1.33 [0.96, 2.00] | 1.35 [1.01, 1.88] | 0.677 | 1.33 [0.96, 1.97] | 1.37 [1.02, 1.97] | 0.471 |
| LDL, mmol/L | 2.42 [1.87, 2.99] | 2.41 [1.87, 3.06] | 0.462 | 2.43 [1.88, 3.00] | 2.39 [1.86, 3.05] | 0.901 |
| HDL, mmol/L | 1.03 [0.83, 1.25] | 1.07 [0.87, 1.30] | 0.003 | 1.05 [0.84, 1.25] | 1.04 [0.84, 1.27] | 0.939 |
| Creatinine, μmol/L | 70.00 [56.60, 91.10] | 69.40 [57.00, 86.40] | 0.231 | 69.00 [56.40, 89.80] | 70.00 [58.10, 87.00] | 0.664 |
| Blood urea nitrogen, mmol/L | 5.00 [3.82, 7.10] | 4.80 [3.76, 6.45] | 0.004 | 5.00 [3.80, 6.90] | 4.98 [3.89, 6.60] | 0.701 |
| eGFR, mL/min | 95.00 [78.40, 107.60] | 98.00 [82.40, 113.00] | <0.001 | 95.30 [80.10, 108.00] | 95.20 [77.80, 111.60] | 0.64 |
| Sodium, mmol/L | 138.30 [136.00, 141.00] | 139.50 [136.90, 141.70] | <0.001 | 138.50 [136.00, 141.00] | 139.00 [136.40, 141.70] | 0.010 |
| Potassium, mmol/L | 3.97 [3.62, 4.30] | 3.99 [3.68, 4.33] | 0.159 | 3.99 [3.65, 4.31] | 3.98 [3.66, 4.37] | 0.714 |
| Calcium, mmol/L | 2.26 [2.11, 2.38] | 2.25 [2.12, 2.37] | 0.998 | 2.26 [2.13, 2.38] | 2.25 [2.12, 2.37] | 0.576 |
| Lactic acid, mmol/L | 1.70 [1.30, 2.40] | 1.74 [1.27, 2.40] | 0.596 | 1.70 [1.30, 2.40] | 1.78 [1.30, 2.33] | 0.732 |
| **Coagulation function** |  |  |  |  |  |  |
| APTT | 30.30 [27.10, 34.30] | 30.70 [27.10, 35.00] | 0.191 | 29.90 [27.10, 34.10] | 30.90 [27.10, 35.40] | 0.064 |
| Prothrombin time, s | 11.60 [10.60, 13.00] | 11.90 [10.70, 13.30] | 0.001 | 11.50 [10.60, 13.10] | 11.70 [10.50, 13.10] | 0.588 |
| Prothrombin activity, % | 96.00 [84.00, 105.00] | 95.00 [83.00, 106.00] | 0.615 | 96.00 [85.00, 105.00] | 95.00 [84.00, 104.20] | 0.134 |
| Thrombin time, s | 17.00 [15.50, 18.30] | 16.80 [15.50, 18.00] | 0.321 | 17.00 [15.50, 18.30] | 16.70 [15.40, 18.10] | 0.079 |
| International normalized ratio | 0.97 [0.89, 1.07] | 0.98 [0.90, 1.09] | 0.03 | 0.96 [0.89, 1.07] | 0.97 [0.89, 1.07] | 0.565 |
| Fibrinogen, mg/L | 3.50 [2.62, 4.54] | 3.24 [2.56, 4.18] | 0.001 | 3.48 [2.60, 4.42] | 3.49 [2.67, 4.41] | 0.496 |
| D-dimer, mg/L | 0.45 [0.25, 0.91] | 0.54 [0.29, 0.95] | 0.003 | 0.44 [0.25, 0.88] | 0.51 [0.26, 0.92] | 0.126 |
| **Cardiac function related indicators** | | | | | | |
| NT-proBNP | 153.20 [48.00, 507.60] | 125.00 [39.00, 459.20] | 0.014 | 149.30 [46.00, 485.00] | 137.80 [42.74, 425.50] | 0.361 |
| hscTnI | 0.03 [0.01, 2.60] | 0.03 [0.01, 3.50] | 0.054 | 0.03 [0.01, 2.40] | 0.03 [0.00, 2.80] | 0.907 |
| CK-MB | 7.59 [1.00, 13.00] | 6.90 [0.87, 12.00] | 0.08 | 7.30 [1.00, 13.00] | 7.40 [0.90, 13.70] | 0.839 |
| **Diabetes related index** |  |  |  |  |  |  |
| Glucose, mmol/L | 6.74 [5.35, 10.50] | 6.10 [5.15, 8.25] | <0.001 | 6.50 [5.27, 10.09] | 6.94 [5.41, 10.56] | 0.043 |
| HbA1c, % | 7.00 [6.00, 8.60] | 6.40 [5.80, 7.60] | <0.001 | 6.90 [5.90, 8.40] | 6.90 [6.00, 8.70] | 0.399 |
| **Outcomes** |  |  |  |  |  |  |
| ARDS | 21 (2.6) | 114 (2.8) | 0.831 | 15 (2.2) | 24 (3.6) | 0.194 |
| Acute kidney injury | 83 (10.2) | 322 (7.8) | 0.033 | 83 (10.3) | 84 (10.4) | 1 |
| Mechanical ventilation | 87 (10.6) | 122 (3.0) | <0.001 | 64 (9.5) | 33 (4.9) | 0.002 |
| ECMO | 10 (1.2) | 12 (0.3) | 0.001 | 6 (0.9) | 6 (0.9) | 1 |
| Renal replacement therapy | 9 (1.1) | 62 (1.5) | 0.463 | 8 (1.2) | 12 (1.8) | 0.499 |

Data were presented as median and interquartile range (Q1-Q3).

Abbreviation: COPD, chronic obstructive pulmonary disease; CKD, chronic kidney disease; LDL, low-density lipoprotein; HDL, high-density lipoprotein; eGFR, estimated glomerular filtration rate; APTT, activated partial thromboplastin time; NT-proBNP, N-terminal pro-B-type natriuretic peptide; hscTnI, hypersensitive cardiac troponin I; CK-MB, creatine kinase isoenzymes; HbA1c, hemoglobin A1c; ECMO, extracorporeal membrane oxygenation; ARDS, acute respiratory distress syndrome**.**

**Supplementary Table S2: Characteristics of Patients with COVID-19 and the Type 2 Diabetes in Metformin and Non-Metformin groups Before and After PSM**

|  | **All patients with diabetes (n=4922)** | | | **PSM 1:1 (n=810)** | | |
| --- | --- | --- | --- | --- | --- | --- |
|  | **Patients with** **metformin (n=466)** | **Patients without metformin (n=4456)** | **P-value** | **Patients with metformin (n=405)** | **Patients without metformin (n=405)** | **P-value** |
| Gender: Male (%) | 246 (52.8) | 2384 (53.5) | 0.807 | 216 (53.3) | 203 (50.1) | 0.399 |
| Age, years | 65.00 [57.00, 71.00] | 67.00 [58.00, 76.00] | <0.001 | 65.00 [57.00, 71.00] | 65.00 [55.00, 74.00] | 0.983 |
| Age ≥65 years (%) | 237 (50.9) | 2569 (57.7) | 0.006 | 215 (53.1) | 207 (51.1) | 0.622 |
| Age＜65 years (%) | 229 (49.1) | 1887 (42.3) | 0.006 | 190 (46.9) | 198 (48.9) | 0.622 |
| Hospitalization time (days) | 13.00 [7.00, 19.00] | 11.00 [7.00, 18.00] | 0.01 | 13.00 [7.00, 20.00] | 12.00 [7.00, 19.00] | 0.190 |
| COVID-19 Severity (n %) |  |  |  |  |  |  |
| Mild | 204 (43.8) | 1618 (36.3) | <0.001 | 175 (43.2) | 187 (46.2) | 0.833 |
| Normal | 155 (33.3) | 1357 (30.5) |  | 136 (33.6) | 126 (31.1) |  |
| Severe | 91 (19.5) | 1155 (25.9) |  | 79 (19.5) | 76 (18.8) |  |
| Critical | 16 (3.4) | 326 (7.3) |  | 15 (3.7) | 16 (4.0) |  |
| **Vital signs** |  |  |  |  |  |  |
| Systolic blood pressure (mmHg) | 128.00 [119.25, 139.00] | 127.00 [115.00, 140.00] | 0.057 | 128.00 [120.00, 139.00] | 128.00 [120.00, 140.00] | 0.86 |
| Diastolic blood pressure (mmHg) | 78.00 [70.00, 86.00] | 79.00 [70.00, 90.00] | 0.058 | 78.00 [70.00, 86.00] | 78.00 [70.00, 85.00] | 0.38 |
| Respiratory rate (times per minute) | 20.00 [19.00, 20.00] | 20.00 [19.00, 20.00] | 0.487 | 20.00 [19.00, 20.00] | 20.00 [19.00, 20.00] | 0.244 |
| Pulse rate (times per minute) | 80.00 [74.00, 89.00] | 80.00 [74.00, 89.00] | 0.855 | 80.00 [74.00, 88.00] | 78.00 [73.00, 88.00] | 0.152 |
| Temperature (°C) | 36.50 [36.30, 36.70] | 36.50 [36.30, 36.70] | <0.001 | 36.50 [36.30, 36.70] | 36.50 [36.30, 36.70] | 0.104 |
| **Origin Comorbidities** |  |  |  |  |  |  |
| Hypertension (%) | 274 (58.8) | 2802 (62.9) | 0.093 | 235 (58.0) | 220 (54.3) | 0.321 |
| Hypercholesterolemia (%) | 210 (45.1) | 2130 (47.8) | 0.282 | 183 (45.2) | 185 (45.7) | 0.944 |
| Coronary heart disease (%) | 96 (20.6) | 1145 (25.7) | 0.019 | 82 (20.2) | 79 (19.5) | 0.860 |
| COPD (%) | 14 (3.0) | 253 (5.7) | 0.021 | 14 (3.5) | 25 (6.2) | 0.101 |
| Heart failure (%) | 54 (11.6) | 410 (9.2) | 0.011 | 47 (11.6) | 40 (9.9) | 0.496 |
| Arrhythmia (%) | 46 (9.9) | 338 (7.6) | 0.097 | 40 (9.9) | 34 (8.4) | 0.542 |
| CKD (%) | 24 (5.2) | 368 (8.3) | 0.023 | 23 (5.7) | 30 (7.4) | 0.394 |
| **Laboratory results** |  |  |  |  |  |  |
| **Routine blood test** |  |  |  |  |  |  |
| Red blood cell count, ×10^12^/L | 4.17 [3.84, 4.55] | 4.12 [3.67, 4.55] | 0.029 | 4.17 [3.84, 4.53] | 4.19 [3.77, 4.53] | 0.931 |
| White cell count, ×10^9^/L | 6.00 [4.79, 7.62] | 6.16 [4.84, 7.90] | 0.131 | 5.93 [4.80, 7.62] | 6.17 [5.10, 7.71] | 0.143 |
| Hemoglobin g/L | 127.00 [115.00, 138.00] | 124.00 [111.00, 137.00] | 0.003 | 127.00 [115.00, 138.00] | 127.00 [114.00, 139.00] | 0.618 |
| Neutrophil count, ×10^9^/L | 3.88 [2.82, 5.27] | 3.98 [2.93, 5.66] | 0.049 | 3.88 [2.79, 5.27] | 3.96 [2.97, 5.38] | 0.299 |
| Monocyte count, ×10^9^/L | 0.40 [0.32, 0.53] | 0.41 [0.31, 0.55] | 0.481 | 0.40 [0.32, 0.54] | 0.40 [0.31, 0.54] | 0.998 |
| Platelet count, ×10^9^/L | 199.00 [162.00, 238.00] | 193.50 [154.00, 236.00] | 0.099 | 127.00 [115.00, 138.00] | 127.00 [114.00, 139.00] | 0.618 |
| **Blood biochemistry** |  |  |  |  |  |  |
| Alanine aminotransferase, U/L | 20.15 [14.15, 31.77] | 20.50 [14.00, 32.73] | 0.798 | 20.00 [14.00, 32.00] | 19.10 [13.50, 30.70] | 0.203 |
| Aspartate aminotransferase, U/L | 20.70 [15.65, 28.00] | 22.00 [16.60, 31.00] | 0.001 | 20.70 [15.10, 28.60] | 21.00 [16.00, 27.40] | 0.914 |
| Lactate dehydrogenase, U/L | 181.00 [150.00, 223.75] | 188.00 [153.00, 249.30] | 0.005 | 181.00 [149.00, 219.90] | 178.00 [148.00, 229.00] | 0.736 |
| Total bilirubin, umol/L | 10.55 [8.10, 14.70] | 10.60 [7.92, 14.60] | 0.887 | 10.64 [8.20, 14.60] | 10.20 [7.80, 14.30] | 0.298 |
| Total protein, g/L | 68.00 [63.60, 72.57] | 67.05 [61.80, 72.00] | 0.001 | 68.00 [63.40, 72.30] | 68.10 [62.20, 73.50] | 0.727 |
| Globulin, g/L | 27.90 [24.70, 31.78] | 27.90 [24.60, 31.70] | 0.744 | 27.90 [24.60, 31.80] | 28.10 [24.70, 31.50] | 0.797 |
| Albumin, g/L | 39.60 [36.00, 43.20] | 38.70 [34.40, 42.80] | 0.001 | 39.40 [35.90, 43.10] | 40.20 [35.40, 44.00] | 0.29 |
| Alkaline phosphatase, U/L | 66.20 [53.50, 82.00] | 72.10 [58.00, 91.23] | <0.001 | 65.90 [53.50, 82.00] | 69.00 [57.20, 86.20] | 0.024 |
| Total cholesterol, mmol/L | 4.16 [3.45, 5.00] | 4.13 [3.45, 4.93] | 0.49 | 4.20 [3.47, 5.03] | 4.25 [3.51, 4.97] | 0.982 |
| Triglyceride, mmol/L | 1.35 [0.99, 1.94] | 1.35 [1.00, 1.90] | 0.953 | 1.35 [0.99, 1.94] | 1.33 [1.02, 1.88] | 0.848 |
| LDL, mmol/L | 2.42 [1.88, 3.06] | 2.41 [1.87, 3.05] | 0.73 | 2.48 [1.87, 3.09] | 2.42 [1.90, 3.07] | 0.852 |
| HDL, mmol/L | 1.07 [0.86, 1.27] | 1.06 [0.86, 1.29] | 0.984 | 1.06 [0.85, 1.26] | 1.08 [0.86, 1.30] | 0.311 |
| Creatinine, μmol/L | 67.00 [57.00, 83.00] | 69.80 [57.00, 88.00] | 0.03 | 67.00 [56.60, 83.00] | 69.00 [56.00, 83.00] | 0.527 |
| Blood urea nitrogen, mmol/L | 4.52 [3.60, 5.90] | 4.87 [3.80, 6.63] | <0.001 | 4.52 [3.60, 5.90] | 4.50 [3.64, 5.83] | 0.796 |
| eGFR, mL/min | 98.70 [85.54, 112.17] | 97.25 [81.38, 112.20] | 0.268 | 98.40 [85.40, 111.00] | 99.20 [84.20, 113.70] | 0.356 |
| Sodium, mmol/L | 139.05 [137.00, 141.88] | 139.20 [136.70, 141.60] | 0.997 | 139.20 [137.00, 141.90] | 139.30 [136.90, 141.70] | 0.784 |
| Potassium, mmol/L | 3.96 [3.67, 4.29] | 3.99 [3.67, 4.33] | 0.232 | 3.96 [3.68, 4.28] | 3.92 [3.59, 4.22] | 0.056 |
| Calcium, mmol/L | 2.29 [2.16, 2.41] | 2.24 [2.12, 2.36] | <0.001 | 2.28 [2.16, 2.41] | 2.28 [2.16, 2.40] | 0.887 |
| Lactic acid, mmol/L | 1.70 [1.20, 2.30] | 1.79 [1.30, 2.40] | 0.086 | 1.70 [1.24, 2.30] | 1.70 [1.23, 2.30] | 0.868 |
| **Coagulation function** |  |  |  |  |  |  |
| APTT | 30.05 [26.70, 34.38] | 30.70 [27.17, 35.00] | 0.053 | 30.10 [26.80, 34.50] | 29.50 [26.90, 33.60] | 0.319 |
| Prothrombin time, s | 11.50 [10.50, 12.80] | 11.90 [10.70, 13.30] | <0.001 | 11.60 [10.50, 12.80] | 11.20 [10.40, 12.60] | 0.158 |
| Prothrombin activity, % | 96.00 [86.00, 105.00] | 95.00 [83.00, 106.00] | 0.408 | 96.00 [85.00, 105.00] | 97.00 [85.00, 106.00] | 0.385 |
| Thrombin time, s | 16.90 [15.40, 18.10] | 16.80 [15.50, 18.10] | 0.808 | 16.80 [15.40, 18.10] | 16.90 [15.40, 18.10] | 0.964 |
| International normalized ratio | 0.96 [0.88, 1.06] | 0.98 [0.90, 1.09] | 0.001 | 0.96 [0.88, 1.06] | 0.93 [0.87, 1.03] | 0.077 |
| Fibrinogen, mg/L | 3.38 [2.56, 4.40] | 3.27 [2.57, 4.21] | 0.388 | 3.37 [2.56, 4.32] | 3.48 [2.64, 4.32] | 0.644 |
| D-dimer, mg/L | 0.36 [0.21, 0.70] | 0.54 [0.29, 0.97] | <0.001 | 0.37 [0.22, 0.72] | 0.46 [0.27, 0.85] | 0.005 |
| **Cardiac function related indicators** | |  |  |  |  |  |
| NT-proBNP | 119.40 [36.49, 336.52] | 131.85 [40.69, 474.62] | 0.202 | 120.60 [38.00, 333.00] | 112.00 [39.00, 420.40] | 0.899 |
| hscTnI | 0.03 [0.01, 2.77] | 0.03 [0.01, 3.40] | 0.149 | 0.03 [0.01, 3.10] | 0.03 [0.00, 2.50] | 0.299 |
| CK-MB | 7.90 [0.76, 13.00] | 7.00 [0.90, 12.03] | 0.685 | 7.90 [0.73, 13.00] | 7.59 [1.06, 14.00] | 0.400 |
| **Diabetes related index** |  |  |  |  |  |  |
| Glucose, mmol/L | 6.36 [5.21, 9.00] | 6.15 [5.17, 8.50] | 0.151 | 6.20 [5.16, 8.88] | 6.13 [5.16, 8.20] | 0.407 |
| HbA1c, % | 6.60 [5.90, 8.17] | 6.50 [5.80, 7.70] | 0.009 | 6.50 [5.90, 8.00] | 6.60 [5.90, 7.90] | 0.830 |
| **Outcomes** |  |  |  |  |  |  |
| ARDS | 3 (0.6) | 132 (3.0) | 0.006 | 3 (0.7) | 6 (1.5) | 0.503 |
| Acute kidney injury | 15 (3.2) | 380 (8.8) | <0.001 | 13 (3.2) | 19 (4.7) | 0.367 |
| Mechanical ventilation | 14 (3.0) | 195 (4.4) | 0.202 | 11 (2.7) | 24 (5.9) | 0.038 |
| ECMO | 3 (0.6) | 19 (0.4) | 0.761 | 3 (0.7) | 6 (1.5) | 0.503 |
| Renal replacement therapy | 0 (0.0) | 71 (1.6) | 0.011 | 0 (0.0) | 1 (0.2) | 1.000 |

Data were presented as median and interquartile range (Q1-Q3).

Abbreviation: COPD, chronic obstructive pulmonary disease; CKD, chronic kidney disease; LDL, low-density lipoprotein; HDL, high-density lipoprotein; eGFR, estimated glomerular filtration rate; APTT, activated partial thromboplastin time; NT-proBNP, N-terminal pro-B-type natriuretic peptide; hscTnI, hypersensitive cardiac troponin I; CK-MB, creatine kinase isoenzymes; HbA1c, hemoglobin A1c; ECMO, extracorporeal membrane oxygenation; ARDS, acute respiratory distress syndrome**.**

**Supplementary Table S3: Characteristics of Patients with COVID-19 and the Type 2 Diabetes in AGIs and Non-AGIs groups Before and After PSM**

|  | **All patients with diabetes (n=4922)** | | | **PSM 1:1 (n=992)** | | |
| --- | --- | --- | --- | --- | --- | --- |
|  | **Patients with AGIs (n=608)** | **Patients without AGIs (n=4314)** | **P-value** | **Patients with AGIs (n=496)** | **Patients without AGIs (n=496)** | **P-value** |
| Gender: Male (%) | 313 (51.5) | 2317 (53.7) | 0.323 | 262 (52.8) | 266 (53.6) | 0.849 |
| Age, years | 67.00 [59.00, 75.00] | 66.00 [58.00, 76.00] | 0.009 | 67.00 [59.00, 75.00] | 68.00 [58.00, 77.00] | 0.99 |
| Age ≥65 years (%) | 368 (60.5) | 2438 (56.5) | 0.068 | 297 (59.9) | 302 (60.9) | 0.795 |
| Age＜65 years (%) | 240 (39.5) | 1876 (43.5) |  | 199 (40.1) | 194 (39.1) | 0.795 |
| Hospitalization time (days) | 14.00 [8.00, 21.00] | 11.00 [7.00, 18.00] | <0.001 | 13.00 [8.00, 20.00] | 12.50 [7.00, 19.00] | 0.168 |
| COVID-19 Severity (n %) |  |  |  |  |  |  |
| Mild | 251 (41.3) | 1571 (36.4) | 0.004 | 202 (40.7) | 217 (43.8) | 0.344 |
| Normal | 196 (32.2) | 1316 (30.5) |  | 162 (32.7) | 165 (33.3) |  |
| Severe | 134 (22.0) | 1112 (25.8) |  | 112 (22.6) | 90 (18.1) |  |
| Critical | 27 (4.4) | 315 (7.3) |  | 20 (4.0) | 24 (4.8) |  |
| **Vital signs** |  |  |  |  |  |  |
| Systolic blood pressure (mmHg) | 128.00 [120.00, 139.00] | 127.00 [114.00, 140.00] | 0.004 | 129.00 [120.00, 140.00] | 128.00 [120.00, 140.00] | 0.594 |
| Diastolic blood pressure (mmHg) | 76.00 [70.00, 84.00] | 80.00 [70.00, 90.00] | <0.001 | 77.00 [70.00, 85.00] | 76.00 [70.00, 83.00] | 0.475 |
| Respiratory rate (times per minute) | 20.00 [19.00, 20.00] | 20.00 [19.00, 20.00] | 0.323 | 20.00 [19.00, 20.00] | 20.00 [19.00, 20.00] | 0.89 |
| Pulse rate (times per minute) | 78.00 [72.00, 88.00] | 80.00 [75.00, 89.00] | 0.001 | 79.00 [72.00, 88.00] | 78.00 [74.00, 86.00] | 0.785 |
| Temperature (°C) | 36.50 [36.30, 36.60] | 36.50 [36.30, 36.77] | <0.001 | 36.50 [36.30, 36.60] | 36.50 [36.30, 36.70] | 0.557 |
| **Origin Comorbidities** |  |  |  |  |  |  |
| Hypertension (%) | 394 (64.8) | 2682 (62.2) | 0.226 | 303 (61.1) | 285 (57.5) | 0.272 |
| Hypercholesterolemia (%) | 303 (49.8) | 2037 (47.2) | 0.243 | 238 (48.0) | 241 (48.6) | 0.899 |
| Coronary heart disease (%) | 158 (26.0) | 1083 (25.1) | 0.675 | 127 (25.6) | 109 (22.0) | 0.205 |
| COPD (%) | 37 (6.1) | 230 (5.3) | 0.501 | 28 (5.6) | 22 (4.4) | 0.468 |
| Heart failure (%) | 75 (12.3) | 389 (9.0) | 0.011 | 59 (11.9) | 69 (13.9) | 0.394 |
| Arrhythmia (%) | 56 (9.2) | 328 (7.6) | 0.193 | 39 (7.9) | 53 (10.7) | 0.155 |
| CKD (%) | 44 (7.2) | 348 (8.1) | 0.53 | 29 (5.8) | 51 (10.3) | 0.014 |
| **Laboratory results** |  |  |  |  |  |  |
| **Routine blood test** |  |  |  |  |  |  |
| Red blood cell count, ×10^12^/L | 4.11 [3.73, 4.54] | 4.13 [3.69, 4.56] | 0.936 | 4.12 [3.74, 4.55] | 4.10 [3.71, 4.47] | 0.179 |
| White cell count, ×10^9^/L | 6.04 [4.98, 7.47] | 6.16 [4.82, 7.96] | 0.084 | 6.02 [5.00, 7.37] | 6.18 [4.87, 7.90] | 0.168 |
| Hemoglobin g/L | 124.00 [113.00, 137.00] | 125.00 [111.00, 137.00] | 0.806 | 125.00 [113.00, 138.00] | 124.00 [110.75, 135.70] | 0.189 |
| Neutrophil count, ×10^9^/L | 3.81 [2.92, 5.17] | 4.00 [2.92, 5.70] | 0.018 | 3.79 [2.91, 5.09] | 3.98 [3.00, 5.70] | 0.041 |
| Monocyte count, ×10^9^/L | 0.41 [0.31, 0.54] | 0.41 [0.31, 0.55] | 0.86 | 0.40 [0.31, 0.53] | 0.40 [0.30, 0.53] | 0.595 |
| Platelet count, ×10^9^/L | 192.00 [156.00, 231.25] | 195.00 [154.00, 236.75] | 0.432 | 191.50 [156.00, 230.00] | 198.00 [156.00, 240.25] | 0.121 |
| **Blood biochemistry** |  |  |  |  |  |  |
| Alanine aminotransferase, U/L | 21.00 [14.00, 32.00] | 20.35 [14.00, 32.60] | 0.961 | 21.00 [14.60, 34.05] | 19.50 [13.80, 32.02] | 0.201 |
| Aspartate aminotransferase, U/L | 21.05 [16.00, 29.00] | 21.80 [16.60, 31.00] | 0.070 | 21.25 [16.30, 29.00] | 21.00 [15.60, 30.00] | 0.6 |
| Lactate dehydrogenase, U/L | 187.00 [155.00, 233.00] | 187.10 [152.40, 249.00] | 0.354 | 184.75 [154.00, 233.32] | 185.25 [148.00, 238.35] | 0.894 |
| Total bilirubin, umol/L | 10.50 [8.00, 15.10] | 10.60 [7.96, 14.50] | 0.784 | 10.70 [8.05, 15.40] | 10.66 [7.88, 14.70] | 0.426 |
| Total protein, g/L | 67.50 [62.58, 72.20] | 67.05 [61.82, 72.00] | 0.184 | 67.40 [62.20, 71.93] | 67.40 [62.30, 71.82] | 0.805 |
| Globulin, g/L | 28.20 [24.70, 31.80] | 27.90 [24.60, 31.70] | 0.394 | 28.10 [24.60, 31.63] | 28.05 [24.78, 32.10] | 0.514 |
| Albumin, g/L | 39.20 [35.10, 42.40] | 38.80 [34.50, 42.90] | 0.500 | 39.30 [35.20, 42.52] | 38.80 [34.85, 42.60] | 0.421 |
| Alkaline phosphatase, U/L | 65.90 [53.00, 82.12] | 72.70 [58.00, 92.00] | <0.001 | 65.00 [53.00, 81.32] | 69.00 [55.58, 85.00] | 0.03 |
| Total cholesterol, mmol/L | 4.10 [3.40, 4.82] | 4.14 [3.46, 4.96] | 0.152 | 4.16 [3.42, 4.89] | 4.07 [3.50, 4.91] | 0.925 |
| Triglyceride, mmol/L | 1.31 [0.98, 1.88] | 1.36 [1.00, 1.90] | 0.245 | 1.33 [0.97, 1.88] | 1.36 [0.98, 1.88] | 0.633 |
| LDL, mmol/L | 2.38 [1.81, 3.03] | 2.42 [1.87, 3.06] | 0.405 | 2.44 [1.85, 3.08] | 2.36 [1.89, 2.98] | 0.266 |
| HDL, mmol/L | 1.03 [0.84, 1.27] | 1.06 [0.87, 1.29] | 0.115 | 1.05 [0.85, 1.29] | 1.05 [0.85, 1.26] | 0.709 |
| Creatinine, μmol/L | 69.90 [57.35, 87.00] | 69.50 [56.70, 87.00] | 0.622 | 70.00 [57.50, 86.93] | 67.00 [55.00, 84.98] | 0.077 |
| Blood urea nitrogen, mmol/L | 5.00 [3.88, 6.50] | 4.80 [3.76, 6.60] | 0.637 | 5.00 [3.84, 6.40] | 4.78 [3.70, 6.53] | 0.366 |
| eGFR, mL/min | 94.95 [80.20, 109.69] | 97.70 [82.00, 112.58] | 0.012 | 95.75 [80.62, 109.95] | 97.88 [81.38, 109.90] | 0.457 |
| Sodium, mmol/L | 139.10 [136.88, 142.00] | 139.20 [136.70, 141.60] | 0.664 | 139.30 [137.00, 142.00] | 139.05 [136.30, 141.93] | 0.404 |
| Potassium, mmol/L | 3.93 [3.61, 4.23] | 4.00 [3.68, 4.34] | 0.001 | 3.93 [3.61, 4.22] | 3.95 [3.61, 4.30] | 0.692 |
| Calcium, mmol/L | 2.26 [2.12, 2.37] | 2.25 [2.12, 2.37] | 0.970 | 2.25 [2.11, 2.37] | 2.26 [2.14, 2.39] | 0.136 |
| Lactic acid, mmol/L | 1.70 [1.30, 2.37] | 1.77 [1.30, 2.40] | 0.494 | 1.70 [1.29, 2.36] | 1.70 [1.30, 2.40] | 0.811 |
| **Coagulation function** |  |  |  |  |  |  |
| APTT | 29.60 [26.50, 34.30] | 30.70 [27.20, 35.00] | 0.005 | 29.60 [26.58, 34.15] | 30.50 [27.10, 34.50] | 0.134 |
| Prothrombin time, s | 11.45 [10.50, 13.00] | 11.90 [10.70, 13.30] | <0.001 | 11.50 [10.50, 13.00] | 11.50 [10.40, 12.80] | 0.621 |
| Prothrombin activity, % | 96.00 [85.00, 104.30] | 95.00 [83.00, 106.00] | 0.612 | 96.15 [85.00, 105.00] | 95.00 [84.10, 104.90] | 0.39 |
| Thrombin time, s | 17.20 [15.60, 18.30] | 16.70 [15.50, 18.00] | 0.012 | 17.20 [15.60, 18.20] | 17.10 [15.50, 18.22] | 0.585 |
| International normalized ratio | 0.96 [0.88, 1.06] | 0.98 [0.90, 1.09] | 0.003 | 0.96 [0.88, 1.06] | 0.94 [0.88, 1.05] | 0.445 |
| Fibrinogen, mg/L | 3.46 [2.60, 4.25] | 3.27 [2.56, 4.22] | 0.209 | 3.44 [2.60, 4.16] | 3.30 [2.53, 4.24] | 0.568 |
| D-dimer, mg/L | 0.42 [0.24, 0.81] | 0.54 [0.29, 0.97] | <0.001 | 0.42 [0.24, 0.80] | 0.42 [0.24, 0.87] | 0.773 |
| **Cardiac function related indicators** | | | | | | |
| NT-proBNP | 140.25 [44.50, 453.05] | 129.00 [39.74, 467.80] | 0.655 | 133.20 [42.78, 405.40] | 136.00 [40.85, 479.78] | 0.597 |
| hscTnI | 0.03 [0.01, 2.50] | 0.03 [0.01, 3.40] | 0.043 | 0.03 [0.01, 2.40] | 0.02 [0.00, 2.22] | 0.202 |
| CK-MB | 7.00 [0.90, 13.00] | 7.00 [0.90, 12.10] | 0.944 | 7.00 [0.93, 13.00] | 7.05 [0.90, 12.62] | 0.934 |
| **Diabetes related index** |  |  |  |  |  |  |
| Glucose, mmol/L | 6.38 [5.23, 8.98] | 6.13 [5.16, 8.51] | 0.045 | 6.32 [5.23, 8.75] | 6.11 [5.12, 8.91] | 0.491 |
| HbA1c, % | 6.60 [5.90, 8.10] | 6.50 [5.80, 7.70] | 0.048 | 6.60 [5.90, 8.10] | 6.50 [5.90, 7.60] | 0.208 |
| **Outcome** |  |  |  |  |  |  |
| ARDS | 6 (1.0) | 129 (3.0) | 0.007 | 4 (0.8) | 11 (2.2) | 0.119 |
| Acute kidney injury | 40 (6.6) | 365 (8.5) | 0.133 | 33 (6.7) | 34 (6.9) | 1 |
| Mechanical ventilation | 37 (6.1) | 172 (4.0) | 0.022 | 26 (5.2) | 32 (6.5) | 0.499 |
| ECMO | 0 (0.0) | 71 (1.6) | 0.003 | 6 (1.2) | 2 (0.4) | 0.287 |
| Renal replacement therapy | 8 (1.3) | 14 (0.3) | 0.002 | 0 (0.0) | 5 (1.0) | 0.073 |

Data were presented as median and interquartile range (Q1-Q3).

Abbreviation: COPD, chronic obstructive pulmonary disease; CKD, chronic kidney disease; LDL, low-density lipoprotein; HDL, high-density lipoprotein; eGFR, estimated glomerular filtration rate; APTT, activated partial thromboplastin time; NT-proBNP, N-terminal pro-B-type natriuretic peptide; hscTnI, hypersensitive cardiac troponin I; CK-MB, creatine kinase isoenzymes; HbA1c, hemoglobin A1c; ECMO, extracorporeal membrane oxygenation; ARDS, acute respiratory distress syndrome**.**

**Supplementary Table S4: Characteristics of Patients with COVID-19 and the Type 2 Diabetes in Sulfonylureas and Non-** **Sulfonylureas groups Before and After PSM**

|  | **All patients with diabetes (n=4922)** | | | **PSM 1:1 (n=450)** | | |
| --- | --- | --- | --- | --- | --- | --- |
|  | **Patients with sulfonylureas (n=256)** | **Patients without sulfonylureas (n=4666)** | **P-value** | **Patients with sulfonylureas (n=225)** | **Patients without sulfonylureas (n=225)** | **P-value** |
| Gender: Male (%) | 128 (50.0) | 2502 (53.6) | 0.286 | 111 (49.3) | 104 (46.2) | 0.571 |
| Age, years | 66.00 [59.00, 74.00] | 66.00 [58.00, 76.00] | 0.904 | 66.00 [59.00, 74.00] | 68.00 [59.00, 77.00] | 0.413 |
| Age ≥65 years (%) | 144 (56.2) | 2662 (57.1) | 0.851 | 128 (56.9) | 137 (60.9) | 0.443 |
| Age＜65 years (%) | 112 (43.8) | 2004 (42.9) | 0.851 | 97 (43.1) | 88 (39.1) | 0.443 |
| Hospitalization time (days) | 14.00 [8.00, 21.00] | 11.00 [7.00, 18.00] | <0.001 | 14.00 [8.00, 21.00] | 12.00 [7.00, 19.00] | 0.049 |
| COVID-19 Severity (n %) |  |  |  |  |  |  |
| Mild | 114 (44.5) | 1708 (36.6) | 0.015 | 185 (82.2) | 177 (78.7) | 0.718 |
| Normal | 79 (30.9) | 1433 (30.7) |  | 10 (4.4) | 15 (6.7) |  |
| Severe | 45 (17.6) | 1201 (25.7) |  | 17 (7.6) | 18 (8.0) |  |
| Critical | 18 (7.0) | 324 (6.9) |  | 13 (5.8) | 15 (6.7) |  |
| **Vital signs** |  |  |  |  |  |  |
| Systolic blood pressure (mmHg) | 128.00 [120.00, 139.00] | 127.00 [115.00, 140.00] | 0.219 | 128.00 [120.00, 139.00] | 128.00 [119.00, 138.00] | 0.900 |
| Diastolic blood pressure (mmHg) | 77.00 [70.00, 85.00] | 79.00 [70.00, 90.00] | 0.001 | 77.00 [70.00, 85.00] | 78.00 [70.00, 85.00] | 0.471 |
| Respiratory rate (times per minute) | 20.00 [19.00, 20.00] | 20.00 [19.00, 20.00] | 0.56 | 20.00 [19.00, 20.00] | 20.00 [19.00, 20.00] | 0.307 |
| Pulse rate (times per minute) | 79.00 [72.75, 89.25] | 80.00 [74.00, 89.00] | 0.527 | 79.00 [74.00, 89.00] | 80.00 [74.00, 88.00] | 0.864 |
| Temperature (°C) | 36.50 [36.30, 36.70] | 36.50 [36.30, 36.70] | 0.142 | 36.50 [36.30, 36.70] | 36.50 [36.30, 36.70] | 0.134 |
| **Origin Comorbidities** |  |  |  |  |  |  |
| Hypertension (%) | 159 (62.1) | 2917 (62.5) | 0.949 | 135 (60.0) | 134 (59.6) | 1 |
| Hypercholesterolemia (%) | 134 (52.3) | 2206 (47.3) | 0.13 | 113 (50.2) | 124 (55.1) | 0.345 |
| Coronary heart disease (%) | 61 (23.8) | 1180 (25.3) | 0.652 | 51 (22.7) | 49 (21.8) | 0.91 |
| COPD (%) | 11 (4.3) | 256 (5.5) | 0.499 | 9 (4.0) | 8 (3.6) | 1 |
| Heart failure (%) | 29 (11.3) | 435 (9.3) | 0.337 | 24 (10.7) | 27 (12.0) | 0.766 |
| Arrhythmia (%) | 21 (8.2) | 363 (7.8) | 0.900 | 15 (6.7) | 30 (13.3) | 0.028 |
| CKD (%) | 19 (7.4) | 373 (8.0) | 0.833 | 14 (6.2) | 12 (5.3) | 0.84 |
| **Laboratory results** |  |  |  |  |  |  |
| **Routine blood test** |  |  |  |  |  |  |
| Red blood cell count, ×10^12^/L | 4.10 [3.74, 4.49] | 4.13 [3.69, 4.56] | 0.727 | 4.08 [3.73, 4.53] | 4.18 [3.77, 4.58] | 0.346 |
| White cell count, ×10^9^/L | 6.02 [5.00, 7.66] | 6.15 [4.82, 7.90] | 0.572 | 6.00 [5.03, 7.68] | 6.29 [4.73, 7.81] | 0.834 |
| Hemoglobin g/L | 123.00 [113.00, 137.25] | 125.00 [111.10, 137.00] | 0.89 | 123.00 [112.00, 138.00] | 126.00 [115.00, 139.00] | 0.355 |
| Neutrophil count, ×10^9^/L | 3.83 [3.05, 5.19] | 3.99 [2.91, 5.63] | 0.345 | 3.83 [3.03, 5.25] | 3.73 [2.73, 5.46] | 0.45 |
| Monocyte count, ×10^9^/L | 0.40 [0.30, 0.53] | 0.41 [0.31, 0.55] | 0.399 | 0.40 [0.30, 0.52] | 0.44 [0.32, 0.58] | 0.04 |
| Platelet count, ×10^9^/L | 203.00 [158.00, 238.25] | 194.00 [154.00, 236.00] | 0.168 | 200.00 [157.00, 235.00] | 199.00 [164.00, 243.00] | 0.557 |
| **Blood biochemistry** |  |  |  |  |  |  |
| Alanine aminotransferase, U/L | 22.00 [13.75, 34.05] | 20.30 [14.00, 32.30] | 0.624 | 21.60 [13.00, 34.00] | 20.30 [14.50, 31.00] | 0.97 |
| Aspartate aminotransferase, U/L | 21.00 [16.00, 28.00] | 21.85 [16.60, 30.90] | 0.266 | 21.00 [16.00, 28.00] | 21.10 [16.60, 29.10] | 0.722 |
| Lactate dehydrogenase, U/L | 181.50 [153.68, 234.25] | 187.50 [153.00, 247.65] | 0.177 | 181.00 [153.00, 228.00] | 182.00 [153.00, 222.00] | 0.974 |
| Total bilirubin, umol/L | 10.85 [8.17, 15.12] | 10.60 [7.92, 14.50] | 0.231 | 10.90 [8.30, 15.20] | 10.10 [7.30, 14.30] | 0.036 |
| Total protein, g/L | 68.90 [64.70, 73.00] | 67.00 [61.80, 72.00] | <0.001 | 68.70 [64.10, 72.90] | 67.50 [62.80, 73.30] | 0.405 |
| Globulin, g/L | 28.60 [25.08, 32.02] | 27.90 [24.60, 31.70] | 0.082 | 28.60 [25.00, 31.80] | 28.00 [24.80, 31.60] | 0.614 |
| Albumin, g/L | 40.05 [36.20, 42.90] | 38.80 [34.50, 42.80] | 0.014 | 40.10 [36.20, 43.00] | 39.80 [35.50, 44.10] | 0.942 |
| Alkaline phosphatase, U/L | 66.00 [54.00, 81.47] | 72.00 [57.62, 91.00] | <0.001 | 66.00 [54.00, 82.00] | 68.00 [53.20, 88.00] | 0.500 |
| Total cholesterol, mmol/L | 4.20 [3.50, 4.89] | 4.13 [3.44, 4.94] | 0.709 | 4.28 [3.62, 4.90] | 4.18 [3.49, 4.95] | 0.678 |
| Triglyceride, mmol/L | 1.40 [1.04, 1.93] | 1.35 [0.99, 1.90] | 0.279 | 1.42 [1.05, 1.98] | 1.38 [1.00, 1.90] | 0.47 |
| LDL, mmol/L | 2.36 [1.90, 3.04] | 2.42 [1.86, 3.05] | 0.883 | 2.43 [1.99, 3.09] | 2.47 [1.95, 3.18] | 0.829 |
| HDL, mmol/L | 1.02 [0.84, 1.30] | 1.06 [0.86, 1.29] | 0.654 | 1.03 [0.86, 1.31] | 0.99 [0.86, 1.25] | 0.172 |
| Creatinine, μmol/L | 66.10 [57.00, 84.40] | 69.80 [57.00, 87.07] | 0.222 | 65.10 [56.80, 83.40] | 69.80 [58.00, 86.40] | 0.213 |
| Blood urea nitrogen, mmol/L | 4.62 [3.70, 5.60] | 4.84 [3.80, 6.60] | 0.01 | 4.70 [3.70, 5.60] | 4.80 [3.50, 6.50] | 0.449 |
| eGFR, mL/min | 97.93 [85.95, 111.39] | 97.40 [81.40, 112.30] | 0.52 | 98.60 [87.00, 112.19] | 97.85 [82.00, 111.00] | 0.439 |
| Sodium, mmol/L | 139.80 [137.60, 142.00] | 139.20 [136.70, 141.60] | 0.032 | 140.00 [137.90, 142.00] | 139.00 [136.20, 141.60] | 0.034 |
| Potassium, mmol/L | 3.92 [3.59, 4.26] | 3.99 [3.67, 4.33] | 0.009 | 3.92 [3.59, 4.28] | 3.98 [3.62, 4.30] | 0.288 |
| Calcium, mmol/L | 2.26 [2.12, 2.39] | 2.25 [2.12, 2.37] | 0.461 | 2.26 [2.13, 2.39] | 2.28 [2.17, 2.39] | 0.311 |
| Lactic acid, mmol/L | 1.69 [1.25, 2.40] | 1.77 [1.30, 2.40] | 0.533 | 1.69 [1.20, 2.32] | 1.70 [1.20, 2.50] | 0.961 |
| **Coagulation function** |  |  |  |  |  |  |
| APTT | 30.10 [26.87, 34.50] | 30.70 [27.10, 34.80] | 0.34 | 29.80 [26.80, 33.90] | 29.50 [26.10, 33.50] | 0.342 |
| Prothrombin time, s | 11.45 [10.40, 12.80] | 11.80 [10.70, 13.30] | 0.002 | 11.40 [10.40, 12.70] | 11.30 [10.50, 12.60] | 0.506 |
| Prothrombin activity, % | 96.70 [84.70, 104.93] | 95.00 [83.00, 106.00] | 0.576 | 97.00 [85.00, 105.00] | 96.00 [85.00, 105.00] | 0.759 |
| Thrombin time, s | 16.80 [15.50, 17.92] | 16.80 [15.50, 18.10] | 0.47 | 16.80 [15.50, 18.00] | 17.10 [15.60, 18.20] | 0.377 |
| International normalized ratio | 0.96 [0.89, 1.06] | 0.98 [0.90, 1.09] | 0.034 | 0.96 [0.89, 1.06] | 0.94 [0.88, 1.05] | 0.374 |
| Fibrinogen, mg/L | 3.38 [2.62, 4.42] | 3.28 [2.56, 4.21] | 0.247 | 3.36 [2.60, 4.27] | 3.23 [2.54, 4.37] | 0.633 |
| D-dimer, mg/L | 0.41 [0.23, 0.82] | 0.53 [0.29, 0.95] | <0.001 | 0.40 [0.22, 0.81] | 0.42 [0.24, 0.84] | 0.329 |
| **Cardiac function related indicators** | | | | | | |
| NT-proBNP | 145.20 [37.83, 359.17] | 129.05 [40.32, 473.70] | 0.576 | 148.00 [38.00, 358.40] | 107.00 [39.00, 425.40] | 0.781 |
| hscTnI | 0.03 [0.00, 2.10] | 0.03 [0.01, 3.40] | 0.032 | 0.03 [0.01, 2.00] | 0.03 [0.01, 2.40] | 0.555 |
| CK-MB | 8.00 [0.92, 14.00] | 7.00 [0.90, 12.10] | 0.113 | 7.60 [0.90, 14.00] | 8.00 [1.20, 14.00] | 0.203 |
| **Diabetes related index** |  |  |  |  |  |  |
| Glucose, mmol/L | 6.27 [5.24, 8.42] | 6.16 [5.17, 8.59] | 0.856 | 6.19 [5.25, 8.10] | 6.22 [5.06, 7.81] | 0.551 |
| HbA1c, % | 6.60 [5.97, 8.00] | 6.50 [5.80, 7.80] | 0.091 | 6.60 [6.00, 7.90] | 6.50 [5.80, 7.80] | 0.285 |
| **Outcome** |  |  |  |  |  |  |
| ARDS | 6 (2.3) | 129 (2.8) | 0.838 | 5 (2.2) | 2 (0.9) | 0.446 |
| Acute kidney injury | 13 (5.1) | 392 (8.4) | 0.077 | 11 (4.9) | 15 (6.7) | 0.544 |
| Mechanical ventilation | 20 (7.8) | 189 (4.1) | 0.006 | 18 (8.0) | 5 (2.2) | 0.010 |
| ECMO | 1 (0.4) | 21 (0.5) | 1 | 1 (0.4) | 2 (0.9) | 1 |
| Renal replacement therapy | 0 (0.0) | 71 (1.5) | 0.086 | 0 (0) | 0 (0) | NA |

Data were presented as median and interquartile range (Q1-Q3).

Abbreviation: COPD, chronic obstructive pulmonary disease; CKD, chronic kidney disease; LDL, low-density lipoprotein; HDL, high-density lipoprotein; eGFR, estimated glomerular filtration rate; APTT, activated partial thromboplastin time; NT-proBNP, N-terminal pro-B-type natriuretic peptide; hscTnI, hypersensitive cardiac troponin I; CK-MB, creatine kinase isoenzymes; HbA1c, hemoglobin A1c; ECMO, extracorporeal membrane oxygenation; ARDS, acute respiratory distress syndrome**.**

**Supplementary Table S5: Characteristics of Patients with COVID-19 and the Type 2 Diabetes in Glinides and Non-Glinides groups Before and After PSM**

|  | **All patients with diabetes (n=4922)** | | | **PSM 1:1 (n=96)** | | |
| --- | --- | --- | --- | --- | --- | --- |
|  | **Patients with glinides (n=57)** | **Patients without glinides (n=4865)** | **P-value** | **Patients with glinides (n=48)** | **Patients without glinides (n=48)** | **P-value** |
| Gender: Male (%) | 30 (52.6) | 2600 (53.4) | 1 | 25 (52.1) | 23 (47.9) | 0.838 |
| Age, years | 66.00 [58.00, 75.00] | 66.00 [58.00, 76.00] | 0.817 | 69.50 [58.00, 80.25] | 68.00 [58.00, 77.25] | 0.921 |
| Age ≥65 years (%) | 30 (52.6) | 2776 (57.1) | 0.591 | 28 (58.3) | 29 (60.4) | 1 |
| Age＜65 years (%) | 27 (47.4) | 2089 (42.9) | 0.591 | 20 (41.7) | 19 (39.6) | 1 |
| Hospitalization time (days) | 16.00 [9.00, 23.00] | 11.00 [7.00, 18.00] | 0.002 | 13.00 [9.00, 20.00] | 18.00 [9.75, 24.00] | 0.151 |
| COVID-19 Severity (n %) |  |  |  |  |  |  |
| Mild | 24 (42.1) | 1798 (37.0) | 0.208 | 20 (41.7) | 22 (45.8) | 0.296 |
| Normal | 19 (33.3) | 1493 (30.7) |  | 17 (35.4) | 15 (31.2) |  |
| Severe | 14 (24.6) | 1232 (25.3) |  | 11 (22.9) | 8 (16.7) |  |
| Critical | 0 (0.0) | 342 (7.0) |  | 0 (0.0) | 3 (6.2) |  |
| **Vital signs** |  |  |  |  |  |  |
| Systolic blood pressure (mmHg) | 130.00 [120.00, 140.00] | 127.00 [115.00, 140.00] | 0.156 | 125.00 [115.75, 136.50] | 130.00 [119.75, 141.25] | 0.159 |
| Diastolic blood pressure (mmHg) | 78.00 [70.00, 86.00] | 79.00 [70.00, 90.00] | 0.318 | 78.00 [70.00, 80.50] | 79.00 [70.00, 89.00] | 0.394 |
| Respiratory rate (times per minute) | 20.00 [18.00, 20.00] | 20.00 [19.00, 20.00] | 0.062 | 20.00 [19.00, 20.00] | 20.00 [18.00, 20.00] | 0.295 |
| Pulse rate (times per minute) | 77.00 [71.00, 81.00] | 80.00 [74.00, 89.00] | 0.005 | 78.00 [72.00, 81.75] | 77.50 [72.00, 81.50] | 0.918 |
| Temperature (°C) | 36.50 [36.30, 36.60] | 36.50 [36.30, 36.70] | 0.048 | 36.50 [36.30, 36.62] | 36.50 [36.30, 36.60] | 0.600 |
| **Origin Comorbidities** |  |  |  |  |  |  |
| Hypertension (%) | 39 (68.4) | 3037 (62.4) | 0.428 | 30 (62.5) | 32 (66.7) | 0.831 |
| Hypercholesterolemia (%) | 26 (45.6) | 2314 (47.6) | 0.873 | 22 (45.8) | 24 (50.0) | 0.838 |
| Coronary heart disease (%) | 8 (14.0) | 1233 (25.3) | 0.072 | 8 (16.7) | 11 (22.9) | 0.608 |
| COPD (%) | 2 (3.5) | 265 (5.4) | 0.728 | 2 (4.2) | 3 (6.2) | 1 |
| Heart failure (%) | 5 (8.8) | 459 (9.4) | 1 | 5 (10.4) | 7 (14.6) | 0.758 |
| Arrhythmia (%) | 6 (10.5) | 378 (7.8) | 0.601 | 6 (12.5) | 6 (12.5) | 1 |
| CKD (%) | 11 (19.3) | 381 (7.8) | 0.003 | 11 (22.9) | 8 (16.7) | 0.608 |
| **Laboratory results** |  |  |  |  |  |  |
| **Routine blood test** |  |  |  |  |  |  |
| Red blood cell count, ×10^12^/L | 3.93 [3.47, 4.29] | 4.13 [3.70, 4.56] | 0.01 | 3.86 [3.37, 4.36] | 3.92 [3.45, 4.28] | 0.812 |
| White cell count, ×10^9^/L | 5.87 [4.42, 6.95] | 6.15 [4.84, 7.90] | 0.073 | 6.17 [5.12, 7.87] | 5.80 [4.42, 6.91] | 0.068 |
| Hemoglobin g/L | 116.00 [106.00, 128.00] | 125.00 [112.00, 137.00] | 0.006 | 113.50 [102.00, 133.25] | 116.00 [104.50, 128.50] | 0.994 |
| Neutrophil count, ×10^9^/L | 3.57 [2.50, 4.75] | 3.98 [2.92, 5.61] | 0.031 | 4.19 [3.06, 7.12] | 3.55 [2.41, 4.76] | 0.016 |
| Monocyte count, ×10^9^/L | 0.44 [0.32, 0.60] | 0.41 [0.31, 0.55] | 0.327 | 0.42 [0.33, 0.55] | 0.42 [0.29, 0.60] | 0.826 |
| Platelet count, ×10^9^/L | 185.00 [161.00, 215.00] | 194.00 [155.00, 236.00] | 0.19 | 174.50 [149.00, 213.00] | 180.50 [160.25, 214.25] | 0.605 |
| **Blood biochemistry** |  |  |  |  |  |  |
| Alanine aminotransferase, U/L | 16.00 [12.00, 24.70] | 20.50 [14.00, 32.80] | 0.005 | 17.70 [13.00, 34.92] | 16.80 [11.88, 25.02] | 0.178 |
| Aspartate aminotransferase, U/L | 19.00 [16.10, 26.00] | 21.70 [16.60, 30.90] | 0.150 | 22.40 [15.78, 32.85] | 19.00 [16.08, 26.00] | 0.387 |
| Lactate dehydrogenase, U/L | 192.00 [158.00, 230.00] | 187.00 [153.00, 246.00] | 0.867 | 196.50 [163.50, 288.25] | 180.50 [151.65, 224.00] | 0.214 |
| Total bilirubin, umol/L | 9.60 [7.90, 13.80] | 10.60 [7.98, 14.60] | 0.402 | 10.40 [7.39, 13.93] | 9.60 [7.10, 13.48] | 0.479 |
| Total protein, g/L | 66.70 [61.60, 73.60] | 67.10 [61.90, 72.10] | 0.597 | 66.45 [61.60, 70.40] | 66.10 [61.10, 72.47] | 0.493 |
| Globulin, g/L | 28.20 [26.00, 32.10] | 27.90 [24.60, 31.70] | 0.765 | 28.00 [25.85, 32.78] | 28.00 [25.68, 32.23] | 0.687 |
| Albumin, g/L | 38.80 [35.50, 43.20] | 38.90 [34.50, 42.80] | 0.487 | 37.90 [31.45, 41.82] | 38.15 [35.65, 42.30] | 0.185 |
| Alkaline phosphatase, U/L | 63.00 [51.10, 82.60] | 72.00 [57.40, 90.50] | 0.055 | 66.50 [51.42, 77.75] | 63.00 [51.00, 82.15] | 0.985 |
| Total cholesterol, mmol/L | 4.00 [3.26, 5.03] | 4.14 [3.45, 4.94] | 0.407 | 4.04 [3.38, 4.56] | 3.96 [3.14, 5.03] | 0.997 |
| Triglyceride, mmol/L | 1.23 [0.93, 1.87] | 1.35 [1.00, 1.90] | 0.391 | 1.20 [0.98, 1.95] | 1.31 [0.94, 1.86] | 0.747 |
| LDL, mmol/L | 2.14 [1.74, 2.95] | 2.41 [1.87, 3.05] | 0.270 | 2.34 [1.81, 2.84] | 2.10 [1.76, 2.96] | 0.846 |
| HDL, mmol/L | 1.07 [0.82, 1.32] | 1.06 [0.86, 1.29] | 0.893 | 1.01 [0.79, 1.27] | 1.02 [0.82, 1.29] | 0.695 |
| Creatinine, μmol/L | 70.00 [59.00, 111.90] | 69.50 [57.00, 87.00] | 0.157 | 79.55 [65.50, 163.60] | 68.65 [58.28, 112.18] | 0.147 |
| Blood urea nitrogen, mmol/L | 5.75 [4.24, 8.68] | 4.80 [3.80, 6.55] | 0.027 | 6.00 [4.45, 10.85] | 5.20 [3.88, 7.80] | 0.237 |
| eGFR, mL/min | 93.80 [57.73, 104.80] | 97.50 [81.94, 112.30] | 0.033 | 96.60 [62.69, 108.08] | 94.40 [57.00, 104.25] | 0.778 |
| Sodium, mmol/L | 140.00 [137.90, 142.20] | 139.20 [136.70, 141.60] | 0.108 | 139.25 [135.00, 142.32] | 140.00 [137.23, 142.35] | 0.359 |
| Potassium, mmol/L | 4.04 [3.62, 4.35] | 3.99 [3.67, 4.32] | 0.885 | 3.99 [3.72, 4.34] | 4.01 [3.60, 4.36] | 0.642 |
| Calcium, mmol/L | 2.26 [2.15, 2.40] | 2.25 [2.12, 2.37] | 0.600 | 2.26 [2.11, 2.40] | 2.26 [2.16, 2.39] | 0.910 |
| Lactic acid, mmol/L | 1.70 [1.30, 2.10] | 1.74 [1.30, 2.40] | 0.281 | 2.19 [1.60, 2.62] | 1.65 [1.27, 2.10] | 0.002 |
| **Coagulation function** |  |  |  |  |  |  |
| APTT | 30.50 [27.50, 35.40] | 30.70 [27.10, 34.80] | 0.646 | 30.05 [25.75, 35.25] | 31.50 [28.10, 37.02] | 0.408 |
| Prothrombin time, s | 11.10 [10.50, 12.50] | 11.80 [10.70, 13.30] | 0.002 | 11.95 [10.47, 14.00] | 11.00 [10.47, 12.60] | 0.045 |
| Prothrombin activity, % | 96.00 [83.00, 108.00] | 95.00 [83.00, 106.00] | 0.548 | 91.00 [76.25, 104.25] | 96.00 [82.30, 106.50] | 0.215 |
| Thrombin time, s | 17.10 [15.30, 17.90] | 16.80 [15.50, 18.10] | 0.932 | 17.20 [16.15, 18.33] | 16.85 [15.10, 17.80] | 0.248 |
| International normalized ratio | 0.92 [0.86, 1.06] | 0.98 [0.90, 1.09] | 0.006 | 1.00 [0.89, 1.13] | 0.91 [0.86, 1.06] | 0.024 |
| Fibrinogen, mg/L | 3.36 [2.50, 4.31] | 3.29 [2.57, 4.22] | 0.890 | 3.50 [2.75, 4.26] | 3.40 [2.50, 4.31] | 0.872 |
| D-dimer, mg/L | 0.48 [0.27, 0.79] | 0.52 [0.29, 0.95] | 0.496 | 0.55 [0.29, 0.90] | 0.48 [0.26, 0.89] | 0.616 |
| **Cardiac function related indicators** | | | | | | |
| NT-proBNP | 222.00 [58.00, 507.60] | 129.10 [40.06, 464.10] | 0.071 | 279.45 [48.25, 2559.35] | 235.00 [61.48, 529.22] | 0.59 |
| hscTnI | 0.03 [0.00, 4.70] | 0.03 [0.01, 3.30] | 0.967 | 0.03 [0.01, 1.63] | 0.03 [0.01, 4.20] | 0.628 |
| CK-MB | 6.70 [0.73, 16.00] | 7.00 [0.90, 12.20] | 0.861 | 7.24 [1.17, 12.00] | 6.55 [0.72, 16.00] | 0.86 |
| **Diabetes related index** |  |  |  |  |  |  |
| Glucose, mmol/L | 6.18 [5.33, 8.42] | 6.17 [5.17, 8.56] | 0.982 | 6.68 [5.39, 8.95] | 6.63 [5.35, 8.73] | 0.817 |
| HbA1c, % | 6.30 [6.00, 7.70] | 6.50 [5.80, 7.80] | 0.923 | 6.40 [5.80, 7.70] | 6.30 [6.00, 7.73] | 0.918 |
| **Outcome** |  |  |  |  |  |  |
| ARDS | 0 (0.0) | 135 (2.8) | 0.386 | 0 (0) | 0 (0) | NA |
| Acute kidney injury | 9 (15.8) | 396 (8.1) | 0.065 | 8 (16.7) | 48 (29.2) | 0.225 |
| Mechanical ventilation | 0 (0.0) | 209 (4.3) | 0.205 | 0 (0.0) | 7 (14.6) | 0.019 |
| ECMO | 1 (1.8) | 21 (0.4) | 0.624 | 1 (2.1) | 0 (0.0) | 1 |
| Renal replacement therapy | 0 (0.0) | 71 (1.5) | 0.719 | 0 (0.0) | 4 (8.3) | 0.125 |

Data were presented as median and interquartile range (Q1-Q3).

Abbreviation: COPD, chronic obstructive pulmonary disease; CKD, chronic kidney disease; LDL, low-density lipoprotein; HDL, high-density lipoprotein; eGFR, estimated glomerular filtration rate; APTT, activated partial thromboplastin time; NT-proBNP, N-terminal pro-B-type natriuretic peptide; hscTnI, hypersensitive cardiac troponin I; CK-MB, creatine kinase isoenzymes; HbA1c, hemoglobin A1c; ECMO, extracorporeal membrane oxygenation; ARDS, acute respiratory distress syndrome**.**

**Supplementary Table S6: Characteristics of Patients with COVID-19 and the Type 2 Diabetes in DPP4 inhibitors and Non-DPP4 inhibitors groups Before and After PSM**

|  | **All patients with diabetes (n=4922)** | | | **PSM 1:1 (n=180)** | | |
| --- | --- | --- | --- | --- | --- | --- |
|  | **Patients with DPP4 inhibitors (n=108)** | **Patients without DPP4 inhibitors (n=4814)** | **P-value** | **Patients with DPP4 inhibitors (n=90)** | **Patients without DPP4 inhibitors (n=90)** | **P-value** |
| Gender: Male (%) | 60 (55.6) | 2570 (53.4) | 0.727 | 49 (54.4) | 47 (52.2) | 0.881 |
| Age, years | 70.50 [59.50, 84.25] | 66.00 [58.00, 75.00] | 0.010 | 70.00 [57.00, 83.50] | 70.00 [58.00, 76.75] | 0.831 |
| Age ≥65 years (%) | 70 (64.8) | 2736 (56.8) | 0.119 | 57 (63.3) | 59 (65.6) | 0.876 |
| Age＜65 years (%) | 38 (35.2) | 2078 (43.2) | 0.119 | 33 (36.7) | 31 (34.4) | 0.876 |
| Hospitalization time (days) | 15.00 [8.00, 25.00] | 11.00 [7.00, 18.00] | 0.002 | 14.00 [7.25, 22.75] | 11.00 [7.25, 19.00] | 0.365 |
| COVID-19 Severity (n %) |  |  |  |  |  |  |
| Mild | 43 (39.8) | 1779 (37.0) | 0.894 | 35 (38.9) | 37 (41.1) | 0.907 |
| Normal | 33 (30.6) | 1479 (30.7) |  | 26 (28.9) | 26 (28.9) |  |
| Severe | 26 (24.1) | 1220 (25.3) |  | 24 (26.7) | 24 (26.7) |  |
| Critical | 6 (5.6) | 336 (7.0) |  | 5 (5.6) | 3 (3.3) |  |
| **Vital signs** |  |  |  |  |  |  |
| Systolic blood pressure (mmHg) | 128.00 [120.00, 139.25] | 127.00 [115.00, 140.00] | 0.101 | 130.00 [123.00, 142.00] | 131.00 [121.00, 141.75] | 0.991 |
| Diastolic blood pressure (mmHg) | 75.00 [69.00, 82.00] | 79.00 [70.00, 90.00] | 0.001 | 77.00 [70.00, 83.75] | 78.00 [70.00, 82.75] | 0.918 |
| Respiratory rate (times per minute) | 20.00 [19.00, 20.00] | 20.00 [19.00, 20.00] | 0.327 | 20.00 [19.00, 20.00] | 20.00 [19.00, 20.00] | 0.191 |
| Pulse rate (times per minute) | 78.00 [73.50, 86.00] | 80.00 [74.00, 89.00] | 0.044 | 78.00 [74.00, 86.00] | 78.00 [70.50, 85.00] | 0.482 |
| Temperature (°C) | 36.50 [36.30, 36.60] | 36.50 [36.30, 36.70] | 0.095 | 36.50 [36.30, 36.60] | 36.50 [36.30, 36.70] | 0.804 |
| **Origin Comorbidities** |  |  |  |  |  |  |
| Hypertension (%) | 83 (76.9) | 2993 (62.2) | 0.003 | 68 (75.6) | 64 (71.1) | 0.613 |
| Hypercholesterolemia (%) | 57 (52.8) | 2283 (47.4) | 0.315 | 44 (48.9) | 52 (57.8) | 0.296 |
| Coronary heart disease (%) | 32 (29.6) | 1209 (25.1) | 0.339 | 26 (28.9) | 24 (26.7) | 0.868 |
| COPD (%) | 10 (9.3) | 257 (5.3) | 0.118 | 9 (10.0) | 5 (5.6) | 0.404 |
| Heart failure (%) | 26 (24.1) | 438 (9.1) | <0.001 | 19 (21.1) | 17 (18.9) | 0.852 |
| Arrhythmia (%) | 13 (12.0) | 371 (7.7) | 0.139 | 9 (10.0) | 14 (15.6) | 0.372 |
| CKD (%) | 14 (13.0) | 378 (7.9) | 0.078 | 12 (13.3) | 10 (11.1) | 0.820 |
| **Laboratory results** |  |  |  |  |  |  |
| **Routine blood test** |  |  |  |  |  |  |
| Red blood cell count, ×10^12^/L | 4.04 [3.62, 4.48] | 4.13 [3.69, 4.55] | 0.295 | 4.08 [3.60, 4.57] | 4.24 [3.84, 4.50] | 0.387 |
| White cell count, ×10^9^/L | 6.06 [4.95, 7.47] | 6.14 [4.83, 7.90] | 0.561 | 5.86 [4.64, 7.27] | 6.40 [5.08, 7.28] | 0.442 |
| Hemoglobin g/L | 122.50 [109.50, 136.00] | 125.00 [112.00, 137.00] | 0.254 | 123.00 [108.00, 138.00] | 128.00 [116.50, 138.75] | 0.170 |
| Neutrophil count, ×10^9^/L | 3.92 [3.07, 5.40] | 3.97 [2.92, 5.60] | 0.813 | 3.90 [3.06, 4.88] | 4.24 [3.16, 5.36] | 0.268 |
| Monocyte count, ×10^9^/L | 0.44 [0.35, 0.56] | 0.41 [0.31, 0.55] | 0.036 | 0.44 [0.35, 0.55] | 0.40 [0.32, 0.50] | 0.109 |
| Platelet count, ×10^9^/L | 194.50 [150.50, 233.25] | 194.00 [155.00, 236.00] | 0.567 | 194.50 [153.75, 229.25] | 179.00 [155.25, 234.75] | 0.740 |
| **Blood biochemistry** |  |  |  |  |  |  |
| Alanine aminotransferase, U/L | 18.00 [12.78, 30.08] | 20.50 [14.00, 32.60] | 0.053 | 17.75 [12.40, 29.52] | 21.45 [15.00, 31.75] | 0.145 |
| Aspartate aminotransferase, U/L | 20.90 [16.25, 27.40] | 21.80 [16.60, 30.90] | 0.192 | 21.00 [16.92, 27.00] | 20.40 [15.93, 28.50] | 0.944 |
| Lactate dehydrogenase, U/L | 186.00 [157.75, 229.75] | 187.00 [153.00, 247.65] | 0.818 | 186.00 [157.00, 229.00] | 184.60 [149.80, 232.00] | 0.866 |
| Total bilirubin, umol/L | 10.90 [7.88, 13.93] | 10.60 [7.98, 14.60] | 0.942 | 11.35 [8.38, 14.52] | 10.75 [8.00, 15.08] | 0.658 |
| Total protein, g/L | 68.25 [65.60, 73.40] | 67.10 [61.90, 72.00] | 0.003 | 68.60 [65.60, 73.40] | 68.15 [62.42, 71.77] | 0.169 |
| Globulin, g/L | 29.50 [26.62, 31.92] | 27.90 [24.50, 31.70] | 0.002 | 29.50 [26.47, 31.90] | 27.40 [24.72, 30.62] | 0.007 |
| Albumin, g/L | 39.50 [36.22, 42.02] | 38.80 [34.50, 42.90] | 0.437 | 39.55 [36.15, 42.48] | 40.40 [36.28, 42.88] | 0.581 |
| Alkaline phosphatase, U/L | 68.00 [55.35, 85.47] | 72.00 [57.40, 90.70] | 0.110 | 67.35 [54.15, 84.50] | 69.00 [57.95, 80.00] | 0.908 |
| Total cholesterol, mmol/L | 4.00 [3.42, 4.48] | 4.14 [3.45, 4.95] | 0.087 | 4.00 [3.44, 4.49] | 3.92 [3.30, 4.72] | 0.605 |
| Triglyceride, mmol/L | 1.25 [0.94, 1.79] | 1.35 [1.00, 1.90] | 0.372 | 1.21 [0.93, 1.77] | 1.39 [1.03, 1.96] | 0.059 |
| LDL, mmol/L | 2.20 [1.77, 2.81] | 2.42 [1.87, 3.06] | 0.068 | 2.32 [1.78, 2.86] | 2.26 [1.75, 2.89] | 0.758 |
| HDL, mmol/L | 1.00 [0.85, 1.22] | 1.06 [0.86, 1.29] | 0.178 | 1.03 [0.85, 1.22] | 1.01 [0.80, 1.23] | 0.463 |
| Creatinine, μmol/L | 72.00 [60.85, 87.00] | 69.40 [57.00, 87.00] | 0.281 | 71.65 [61.10, 84.45] | 70.95 [58.85, 90.70] | 0.811 |
| Blood urea nitrogen, mmol/L | 5.10 [3.83, 6.43] | 4.80 [3.80, 6.60] | 0.747 | 4.89 [3.82, 6.38] | 4.98 [3.43, 6.36] | 0.759 |
| eGFR, mL/min | 92.75 [79.95, 107.53] | 97.50 [81.80, 112.38] | 0.061 | 93.65 [80.40, 108.57] | 95.90 [79.38, 110.85] | 0.928 |
| Sodium, mmol/L | 138.00 [136.00, 140.93] | 139.30 [136.70, 141.60] | 0.004 | 138.55 [136.00, 141.00] | 139.70 [137.50, 142.00] | 0.017 |
| Potassium, mmol/L | 3.94 [3.61, 4.26] | 3.99 [3.67, 4.32] | 0.304 | 3.94 [3.65, 4.26] | 3.96 [3.75, 4.24] | 0.729 |
| Calcium, mmol/L | 2.28 [2.10, 2.43] | 2.25 [2.12, 2.37] | 0.190 | 2.28 [2.10, 2.44] | 2.30 [2.14, 2.40] | 0.899 |
| Lactic acid, mmol/L | 1.65 [1.28, 2.40] | 1.74 [1.30, 2.40] | 0.722 | 1.60 [1.22, 2.48] | 1.80 [1.40, 2.40] | 0.524 |
| **Coagulation function** |  |  |  |  |  |  |
| APTT | 29.85 [26.28, 35.50] | 30.70 [27.10, 34.80] | 0.626 | 29.25 [26.20, 34.60] | 28.60 [25.95, 33.35] | 0.442 |
| Prothrombin time, s | 11.20 [10.30, 12.53] | 11.80 [10.70, 13.30] | 0.001 | 11.20 [10.30, 12.47] | 11.10 [10.03, 13.17] | 0.709 |
| Prothrombin activity, % | 94.50 [81.92, 103.00] | 95.00 [83.05, 106.00] | 0.469 | 96.00 [83.00, 104.00] | 96.00 [86.00, 103.00] | 0.91 |
| Thrombin time, s | 17.20 [15.40, 18.40] | 16.80 [15.50, 18.10] | 0.367 | 17.00 [15.43, 18.40] | 17.60 [16.15, 18.60] | 0.124 |
| International normalized ratio | 0.94 [0.86, 1.06] | 0.98 [0.90, 1.09] | 0.02 | 0.93 [0.86, 1.03] | 0.94 [0.86, 1.10] | 0.544 |
| Fibrinogen, mg/L | 3.42 [2.73, 4.36] | 3.29 [2.56, 4.22] | 0.372 | 3.38 [2.74, 4.32] | 3.38 [2.54, 4.36] | 0.923 |
| D-dimer, mg/L | 0.47 [0.25, 0.82] | 0.53 [0.29, 0.95] | 0.357 | 0.48 [0.24, 0.80] | 0.38 [0.25, 0.81] | 0.812 |
| **Cardiac function related indicators** | | | | | | |
| NT-proBNP | 126.30 [57.50, 352.08] | 130.00 [40.00, 468.85] | 0.475 | 119.10 [60.42, 343.55] | 106.15 [32.08, 601.57] | 0.564 |
| hscTnI | 0.02 [0.00, 2.12] | 0.03 [0.01, 3.30] | 0.076 | 0.02 [0.00, 2.64] | 0.02 [0.00, 2.50] | 0.664 |
| CK-MB | 7.00 [1.39, 15.00] | 7.00 [0.90, 12.10] | 0.100 | 6.10 [1.38, 14.15] | 7.00 [0.78, 13.00] | 0.367 |
| **Diabetes related index** |  |  |  |  |  |  |
| Glucose, mmol/L | 6.65 [5.52, 10.16] | 6.15 [5.17, 8.52] | 0.007 | 6.60 [5.51, 10.05] | 6.48 [5.29, 10.62] | 0.688 |
| HbA1c, % | 6.80 [6.00, 7.90] | 6.50 [5.80, 7.80] | 0.194 | 6.70 [6.00, 7.88] | 6.40 [5.80, 7.83] | 0.346 |
| **Outcome** |  |  |  |  |  |  |
| ARDS | 1 (0.9) | 134 (2.8) | 0.384 | 1 (1.1) | 1 (1.1) | 1 |
| Acute kidney injury | 10 (9.3) | 395 (8.2) | 0.828 | 8 (8.9) | 6 (6.7) | 0.781 |
| Mechanical ventilation | 4 (3.7) | 205 (4.3) | 0.967 | 3 (3.3) | 9 (10.0) | 0.135 |
| ECMO | 1 (0.9) | 21 (0.4) | 0.98 | 0 (0.0) | 1 (1.1) | 1 |
| Renal replacement therapy | 1 (0.9) | 70 (1.5) | 0.962 | 1 (1.1) | 1 (1.1) | 1 |

Data were presented as median and interquartile range (Q1-Q3).

Abbreviation: COPD, chronic obstructive pulmonary disease; CKD, chronic kidney disease; LDL, low-density lipoprotein; HDL, high-density lipoprotein; eGFR, estimated glomerular filtration rate; APTT, activated partial thromboplastin time; NT-proBNP, N-terminal pro-B-type natriuretic peptide; hscTnI, hypersensitive cardiac troponin I; CK-MB, creatine kinase isoenzymes; HbA1c, hemoglobin A1c; ECMO, extracorporeal membrane oxygenation; ARDS, acute respiratory distress syndrome**.**

**Supplementary Table S7: Hazard Ratios for Secondary Outcomes between Patients with Insulin, Metformin or AGIs**

|  | Patients with insulin | | Patients with metformin | | Patients with AGIs | |
| --- | --- | --- | --- | --- | --- | --- |
| Secondary outcomes | Adjusted HR (95% CI) | P-value | Adjusted HR (95% CI) | P-value | Adjusted HR (95% CI) | P-value |
| ARDS | 1.39 (0.85-2.28) | 0.188 | 0.28 (0.08-0.96) | 0.043 | 0.38 (0.15-0.94) | 0.037 |
| Acute kidney injury | 1.25 (0.96-1.63) | 0.100 | 0.45 (0.26-0.77) | 0.004 | 0.85 (0.58-1.25) | 0.418 |
| Invasive mechanical ventilation | 4.12 (3.03-5.59) | <0.001 | 0.46 (0.26-0.81) | 0.007 | 0.81 (0.53-1.22) | 0.308 |
| ECMO | 2.39 (0.89-6.42) | 0.084 | 0.78 (0.21-2.91) | 0.710 | 2.61 (0.89-7.67) | 0.080 |

The results of cox regression analysis were shown in adjusted HR (95% CI).Abbreviation: ECMO, extracorporeal membrane oxygenation; ARDS, acute respiratory distress syndrome**.**

**Supplementary Table S8:** **Hazard Ratios for Secondary Outcomes between Patients with Sulfonylureas, Glinides or DPP4 inhibitors**

|  | Patients with sulfonylureas | | Patients with glinides | | Patients with DPP4 inhibitors | |
| --- | --- | --- | --- | --- | --- | --- |
| Secondary outcomes | Adjusted HR (95% CI) | P-value | Adjusted HR (95% CI) | P-value | Adjusted HR (95% CI) | P-value |
| ARDS | 2.19 (0.87-5.5) | 0.097 | NA | NA | 0.63 (0.08-4.89) | 0.656 |
| Acute kidney injury | 0.90 (0.50-1.63) | 0.733 | 1.15 (0.56-2.34) | 0.704 | 0.58 (0.29-1.15) | 0.118 |
| Invasive mechanical ventilation | 1.79 (1.07-2.99) | 0.026 | NA | NA | 0.35 (0.13-0.97) | 0.043 |
| ECMO | 0.40 (0.05-3.14) | 0.382 | 1.36 (0.17-10.73) | 0.770 | 0.55 (0.07-4.47) | 0.579 |

The results of cox regression analysis were shown in adjusted HR (95% CI). Abbreviation: ECMO, extracorporeal membrane oxygenation; ARDS, acute respiratory distress syndrome**.**

NA means there was no patient with the secondary outcomes.
